# Novel Wet Electrospinning Inside a Reactive Pre-Ceramic Gel to Yield Advanced Nanofiber-Reinforced Geopolymer Composites

**DOI:** 10.3390/polym14193943

**Published:** 2022-09-21

**Authors:** Yunzhi Xu, Ping Guo, Ange-Therese Akono

**Affiliations:** 1 Department of Civil and Environmental Engineering, Northwestern University, Evanston, IL 60208, USA; 2 Department of Mechanical Engineering, Northwestern University, Evanston, IL 60208, USA

**Keywords:** wet electrospinning, geopolymer, nanofibers, fiber-reinforced ceramic

## Abstract

Electrospinning is a versatile approach to generate nanofibers in situ. Yet, recently, wet electrospinning has been introduced as a more efficient way to deposit isolated fibers inside bulk materials. In wet electrospinning, a liquid bath is adopted, instead of a solid collector, for fiber collection. However, despite several studies focused on wet electrospinning to yield polymer composites, few studies have investigated wet electrospinning to yield ceramic composites. In this paper, we propose a novel in-situ fabrication approach for nanofiber-reinforced ceramic composites based on an enhanced wet-electrospinning method. Our method uses electrospinning to draw polymer nanofibers directly into a reactive pre-ceramic gel, which is later activated to yield advanced nanofiber-reinforced ceramic composites. We demonstrate our method by investigating wet electrospun Polyacrylonitrile and Poly(ethylene oxide) fiber-reinforced geopolymer composites, with fiber weight fractions in the range 0.1–1.0 wt%. Wet electrospinning preserves the amorphous structure of geopolymer while changing the molecular arrangement. Wet electrospinning leads to an increase in both the fraction of mesopores and the overall porosity of geopolymer composites. The indentation modulus is in the range 6.76–8.90 GPa and the fracture toughness is in the range 0.49–0.76 MPa$\sqrt{m}$ with a clear stiffening and toughening effect observed for Poly(ethylene oxide)-reinforced geopolymer composites. This work demonstrates the viability of wet electrospinning to fabricate multifunctional nanofiber-reinforced composites.

## 1. Introduction

Electrospinning is an efficient and versatile method for preparing nanofibers and has been extensively investigated in various engineering fields such as tissue engineering [1,2,3], energy conservation [4,5] and soft electronics [6,7]. The electrospinning method makes use of an electrostatic force to draw a polymer solution or a polymer melt into an ultrafine jet. Before reaching the collector, the electrically charged jet goes through solvent evaporation or solidification, until it is finally deposited on the grounded collector. The electrostatic repulsion along the charged jet causes elongation and thinning of the jet, resulting in the formation of fibers with nanometer-scale diameters [8]. Although polymers are the materials most commonly used in electrospinning, a wide range of materials, including ceramics, composites, and semiconductors, are also applicable in electrospinning to produce nanofibers. Meanwhile, various forms of nanofiber assemblies are achievable through the electrospinning process. Ultraporous fibrous membranes with interconnected nanopores and uniform pore distribution can be obtained through conventional electrospinning, which has been widely applied in liquid and gas filtration [9]. Aligned fiber meshes were fabricated using rotating mandrels, parallel electrodes, and an array of counter-electrodes, and can be used to design laminated composites with a controlled orientation of fiber reinforcements [10]. In addition, fiber yarns were realized through a median coagulation bath, ring collectors, and multiple spinneret setups [10], to facilitate the design of high-performance fabrics, bio-matrix composites, and medical prostheses [11]. Thus far, traditional electrospinning has been primarily investigated to yield truss-like structures. For example, Mozaffari and Gashti [12,13] formulated various processing routes that rely on traditional electrospinning to synthesize crosslinked gelatin nanofiber scaffolds for skin-tissue engineering applications. They showed that traditional electrospinning is suitable to yield 2D nanostructured scaffolds with increased biofunctionality. Almasian et al. [14] produced superhydrophobic PAN nanofiber mats using electrospinning for fog-harvesting applications. They deposited an un-uniform layer of a functionalization compound on the surface of electrospun nanofibers to modify the surface roughness and pore-size distribution. These studies have shown that electrospinning is adequate to yield nanostructures with high functionality, yet the mechanical performance is not explored, suggesting that those materials produced are not adequate for load-bearing applications.

Electrospinning offers great potential for the in-situ production and integration of nanofibers in composite manufacturing. Electrospun fibers usually possess exceptional properties such as controlled chemical composition, high porosity, large surface area and great mechanical strength; these properties are favored in reinforcing materials to enhance performance. However, due to the intricate entanglement of electrospun fibers, it is extremely challenging to achieve a uniform dispersion of electrospun nanofibers in bulk materials. Therefore, incorporating electrospun fibers as reinforcing fillers in composites, such as in polymer-based materials, has so far been mainly achieved through dip-coating or film-stacking methods [15]. For instance, Beckermann et al. [16] used electrospun nylon nanofiber mats as interleaved layers to toughen the epoxy matrix and the produced electrospun nylon nanofiber-reinforced carbon/epoxy composites exhibited a 156% and 69% increase in Mode I and Mode II interlaminar fracture toughness, respectively. Moreover, Wu et al. [17] fabricated poly(methylmethacrylate) (PMMA) composite films reinforced with randomly and uniaxially aligned polyacrylonitrile (PAN) nanofiber mats prepared by electrospinning. The mechanical properties of the composite films were found to steadily improve with the nanofiber content, and they were dependent on the fiber orientation. The composite film containing uniformly aligned nanofibers displayed a 40% increase in the tensile strength and a 30% increase in the Young’s modulus compared to that with randomly organized fibers.

Despite benefits in terms of mechanical enhancements, embedding electrospun fiber mats in composites is a limited approach, which does not fully leverage the potential of isolating individual electrospun nanofibers within bulk materials. The compact nature of the non-woven nanofiber mats obtained from conventional electrospinning substantially diminishes the efficiency of electrospun nanofibers and limits their contribution in reinforcing composites. Molnar et al. [18] investigated the tensile strength of a single electrospun polyamide-6 fiber and reported that the tensile strength of the nanofiber mat is just a fraction, 38.6%, of the tensile strength of a single electrospun nanofiber. In addition, Papkov et al. [19] studied the effect of size on the mechanical properties and the structures of single electrospun PAN nanofibers. As the diameter of the single PAN fiber reduced to around 100 nm, the elastic modulus, true strength and toughness of the single nanofiber increased to 48 GPa, 1.75 GPa, and 605 MPa, respectively. These findings suggest that significant gains in mechanical properties can be achieved by depositing single nanofibers in bulk materials. Yet, novel manufacturing methods are needed to integrate individual electrospun nanofibers within bulk materials.

In recent years, wet electrospinning has been introduced as a more efficient way to deposit isolated fibers in bulk materials. In the wet-electrospinning process, a liquid bath is chosen to collect the electrospun fibers, instead of a solid collector. Yokoyama et al. [20] demonstrated the ability of wet electrospinning to produce 3D spongiform poly (glycolic acid) (PGA) structures with porosity up to 96.7%. Sonseca et al. [21] also reported a 12% increase in the open porosity of 3D scaffolds fabricated using wet electrospinning compared to 2D scaffolds using conventional electrospinning. Meanwhile, the tensile strength of the 3D scaffold was lower than that of 2D scaffold, which was attributed to the highly coiled and ultra-porous structure produced by wet electrospinning. Lin et al. [22] developed an effective approach to fabricate highly compressible 3D nanofibrous aerogels using a graphene oxide aqueous suspension as the liquid-bath collector during the electrospinning of PAN fibers. The produced aerogels exhibited enhanced mechanical strength compared to those using water collection, as well as structural robustness in sustaining large cyclic compressive strain. The open porous structure also allowed a high absorption capacity for different types of oil, which promised significant benefits for future applications in pollutants treatment.

These preliminary studies of wet electrospinning inside a reactive gel calls for more advanced studies to understand the factors affecting the microstructure and performance of composites manufactured via wet electrospinning. An important aspect is electrospinning using a colloidal reactive pre-ceramic bath as a liquid collector. Such an approach is important to yield advanced multiscale ceramic composites.

In a previous work [23], using wet electrospinning, we successfully deposited electrospun Poly(ethylene oxide) (PEO) fibers directly into a potassium silicate solution to yield fiber-reinforced geopolymers. We observed a great bonding between the electrospun fibers and the ceramic matrix. The electrospun fibers also exhibited toughening mechanisms such as crack bridging, resulting in an enhancement of indentation modulus and indentation hardness by 29% and 22%, respectively. However, one limitation of our previous work is the lack of a fundamental understanding of the chemistry of the fabricated geopolymer composites, as well as a systematic investigation of the influence of electrospinning polymers and liquid-bath materials on the final behavior of the materials. Therefore, in this paper, we seek to understand the influence of wet electrospinning on the microstructure, chemistry and mechanical properties of the resulting fiber-reinforced ceramics. We focus on geopolymer precursors as liquid collectors and investigate the physical, chemical, microstructural, and mechanical properties of electrospun fiber-reinforced geopolymer nanocomposites. This article is organized as follows: first our manufacturing process is detailed; then our characterization methods are explained; finally, our results are presented and then discussed.

## 2. Materials and Methods

### 2.1. Wet-Electrospinning Experiments

**Figure 1:**
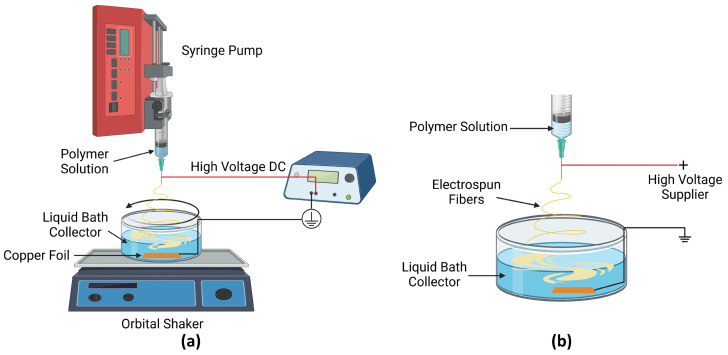
The schematic of experimental setup for wet electrospinning of polymer nanofibers: (**a**) Experimental setup. (**b**) Schematic between the needle and the liquid bath collector. The polymer solution is varied between Polyacrylonitrile (PAN) and Poly(ethylene oxide) (PEO) to investigate the effect of polymer type. The liquid-bath collector is varied between DI water and water glass to investigate the effect of collection bath. The orbital shaker was employed to agitate the liquid bath for fiber-dispersion purposes.

Figure 1 displays a schematic of the wet-electrospinning setup used in this study. The wet-electrospinning technique used a liquid bath as the collector of the polymer fibers. The prepared polymer solutions were loaded in a 6-mL plastic syringe with a stainless-steel needle (gauge 21) and placed on a vertically situated syringe pump (NE-300, New Era Pump Systems, Farmingdale, NY, USA). Positive DC high voltage was supplied by a high-voltage amplifier (Trek 10/10B-HS, Advanced Energy, Denver, CO, USA) and applied to the needle. The applied voltage was varied to obtain stable and continuous fiber generation, in the range of 8–11 kV. The produced nanofibers were collected in a liquid-bath collector with a grounded copper foil inserted at the bottom of the bath. The distance between the needle tip to the surface of the liquid bath was altered based on the applied voltage to achieve a 100 V/mm electric field. During electrospinning, the polymer solution was dispensed at a feed rate of 1 mL/h. The liquid-bath collector was placed on an orbital shaker running with a relative centrifugal force (RCF) of 1 g to both expedite fiber solidification and facilitate fiber dispersion inside the liquid bath.

Polyacrylonitrile (PAN) and Poly(ethylene oxide) (PEO) were selected as nanofiber materials due to their low toxicity and easy handleability. To electrospin PAN fibers, a 14 wt% PAN solution was prepared by dissolving PAN powder (M_w_ = 150,000 g/mol; Sigma-Aldrich, St. Louis, MI, USA) in dimethylformamide (DMF) (Fisher Chemical, Waltham, MA, USA) using a magnetic stirrer and stirring at ambient temperature for 12 h until full dissolution. For PEO-fiber electrospinning, a 20 wt% PEO solution was prepared by dissolving PEO powder (M_w_ = 100,000 g/mol; Sigma-Aldrich) in deionized water and stirring at 30 °C for 12 h to ensure full dissolution.

### 2.2. Synthesis of Geopolymer Nanocomposites Reinforced with Electrospun Fibers

**Figure 2:**
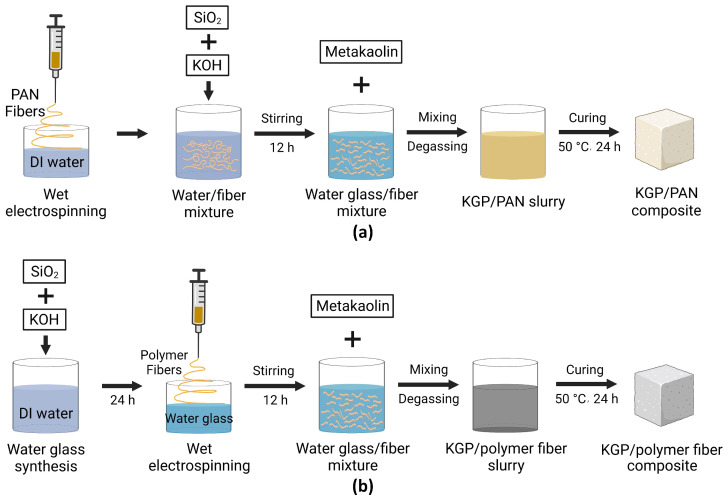
Synthesis process of electrospun-fiber-reinforced geopolymer composites: (**a**) Using DI water as the liquid-bath collector. (**b**) Using the waterglass solution as the liquid-bath collector.

Metakaolin-based potassium geopolymer nanocomposites were manufactured with electrospun PAN and PEO fiber reinforcement, respectively. The reference geopolymer matrix, KGP, is the metakaolin-based potassium geopolymer of chemical formula K${}_{2}$O· Al${}_{2}$O${}_{3}$· 4 SiO${}_{2}$· 11 H${}_{2}$O [24]. The manufacturing process of the electrospun-fiber-reinforced geopolymer nanocomposites is illustrated in Figure 2. Two synthesis procedures were considered, based on the different collection baths involved.

The first procedure employed deionized water as the liquid-bath collector during electrospinning, as shown in Figure 2a. This procedure is limited to water-insoluble polymers, namely, PAN in this paper. First, 27.61 g of deionized (DI) water was used to collect electrospun PAN fibers. Second, 19.87 g of potassium hydroxide pellets and 18.43 g of fumed silica were added to the water/fiber mixture and stirred using a magnetic stirrer for 12 h to form a potassium silicate (waterglass) solution containing uniformly dispersed electrospun PAN fibers. Afterward, the waterglass and fiber mixture was combined with 34.08 g of synthetic metakaolin using a planetary centrifugal mixer (ARE-310, THINKY, Laguna Hills, CA, USA) at 1200 rpm for 5 min and degassed at 1400 rpm for 3 min to produce the fresh KGP/PAN composite slurry. The slurry was then cured at 50 °C on an orbital shaker running at 1 g RCF for 24 h to form the hardened geopolymer nanocomposite samples.

Figure 2b shows a second protocol that uses the waterglass solution as the liquid-bath collector. First, the waterglass solution was synthesized by mixing 27.61 g of DI water with 19.87 g of potassium hydroxide pellets and 18.43 g of fumed silica using a magnetic stirrer. The mixture was then left on an orbital shaker running at 0.13 g RCF for 24 h to ensure the full dissolution of fumed silica. Second, the waterglass was adopted as the liquid-bath collector for the polymer fibers during the wet-electrospinning process. Both PEO and PAN can be collected using the waterglass solution regardless of their water solubility. After wet electrospinning, the waterglass and fiber mixture were stirred with a magnetic stirrer at 800 rpm for 12 h to achieve a uniform fiber dispersion inside the waterglass. The mixture was then mixed with 34.08 g of metakaolin using the same protocol as the first procedure and cured at 50 °C for 24 h to form the final product.

The different electrospun-fiber-reinforced geopolymer nanocomposites synthesized are listed in Table 1. Two types of polymer reinforcement were employed: PAN and PEO. Two liquid-bath collectors were adopted: DI water and waterglass for PAN-reinforced geopolymer composites and waterglass for PEO-reinforced geopolymer composites. Three values of the weight ratio of electrospun polymer fibers were considered, namely, 0.1 wt%, 0.5 wt%, and 1 wt%, for each type of samples. In total, nine composites were synthesized. In the following sections, PAN fiber-reinforced geopolymer using water collection will be referred to as KGP/PAN-W, PAN fiber-reinforced geopolymer using waterglass collection will be referred to as KGP/PAN-WG, and PEO fiber-reinforced geopolymer using waterglass collection will be KGP/PEO-WG.

### 2.3. Material Characterization

#### 2.3.1. Scanning Electron Microscopy

The microstructure of the hardened electrospun-fiber-reinforced geopolymer nanocomposites was characterized using environmental scanning electron microscopy (ESEM). ESEM analysis was performed using an environmental scanning electron microscope (Quanta 650, FEI, Hillsboro, OR, USA) equipped with a backscattered electron detector. The samples were imaged in low-vacuum mode with an accelerating voltage of 10 kV and a working distance of 10 mm. Digital image analysis was conducted based on a 10 × 10 grid SEM images of the cross-section of the samples with a magnification of 1000×. A quantitative analysis of fiber morphology and fiber distribution was performed using digital image analysis in the programming software Python and using the image processing software ImageJ. Specifically, the variational Bayesian Gaussian mixture model was applied to perform a deconvolution based on the histogram of the image grayscale and identify the appropriate threshold value for image segmentation [25]. The fiber area and fiber thickness and fiber fraction were then calculated based on segmented images.

#### 2.3.2. XRD and FTIR

The chemistry of the geopolymer nanocomposite synthesized was investigated using powder X-ray diffraction (XRD) and Fourier-transform infrared spectroscopy (FTIR). Before XRD and FTIR testing, the bulk samples were ground into fine powders using a McCrone (Westmont, IL, USA) XRD mill to a final fineness of less than 1 μm. XRD patterns were collected using an automated X-ray diffractometer (Ultima IV, Rigaku, Tokyo, Japan) equipped with a Cu*K*α source (λ = 1.5406 nm). Tests were conducted at a voltage of 40 kV, a current of 30 mA, and at Bragg angles (2θ) ranging from 5° to 70°. The scan speed was 0.5°/min and the step size was 0.1° [26]. The phase identification was performed using Jade software via whole pattern fitting of X-ray data [27].

FTIR spectra were obtained by an FTIR spectrometer (Nicolet iS50, Thermo Scientific, Waltham, MA, USA) in transmission mode using the KBr method [28]. KBr powder was mixed with the sample powder at a weight ratio of 100:1. The powder mixture was then pressed into pellets for FTIR testing. The FTIR analysis was conducted in absorbance mode from 4000 to 400 cm${}^{-1}$ at a resolution of 4 cm${}^{-1}$ and 64 scans per spectrum.

#### 2.3.3. Micro-CT

The 3D internal structure of the electrospun-fiber-reinforced geopolymer nanocomposites was examined using micro–computed tomography (Micro-CT or μCT). Before micro-CT scanning, the samples were cut into cuboids with a 5 mm × 5 mm square cross-section. The sample size was selected to allow a balance between a representative volume of the sample and the desired resolution, 4 μm in all scanning. The Micro-CT scanning was performed with a 3D X-ray scanner (MicroXCT-200, Xradia, Pleasanton, CA, USA) with a Tungston (W) X-ray tube anode. The specimen was fixed vertically about the specimen’s longitudinal axis. The area of observation was adjusted to a 4 mm diameter circle centered in the middle of the sample stripe. The samples were scanned under a tube voltage of 60 kV and a tube current of 166 μA. For each sample scanning, 900 projections were acquired to cover 360 degrees rotation. The exposure time for each projection was 5 s. The resulting voxel size was 4 μm × 4 μm × 4 μm.

For each sample, 1000 cross-sectional images were reconstructed from the Micro-CT projections. To remove the effect of scanning noise, digital image analysis was conducted on a 500 × 500 × 500 voxel cube centered in the core of the 3D volume scanned, which represented the real size of a 2 mm cube sample volume. The Micro-CT images were first denoised using a Gaussian filter and segmented based on the deconvolution process. Then, an open source FIJI plugin BoneJ [29] was employed to analyze the Micro-CT images in order to achieve a quantitative characterization of the internal structure of the samples. During image analysis, the fiber phase and the porous phase was identified based on the sphericity of the particles [30]. A deconvolution of the sphericity distribution was performed to identify the threshold between the fiber phase and the pore phase.

#### 2.3.4. Mercury Intrusion Porosimetry

The porosity and the pore-size-distribution analyses of the geopolymer nanocomposites were conducted using mercury intrusion porosimetry (MIP). The principle of mercury intrusion porosimetry is to force the intrusion of mercury into the pores of a material under stringently controlled pressures. The relationship between the pore diameter and pressure is described by [31]:

(1) $$\begin{array}{c} d=\frac{-4\gamma cos(\theta )}{P} \end{array}$$

Here, *d* is the pore diameter, γ is the surface tension of mercury, θ is the contact angle of mercury, and *P* is the applied pressure. The pore-size distribution is determined from the volume intruded at each pressure increment and the total porosity is determined from the total volume intruded.

The MIP analysis was performed using a mercury porosimeter (AutoPore V 9605, Micromeritics, Norcross, GA, USA) with a maximum pressure limit of 33,000 psi and resolution of 0.165 psi. The equipment can detect pore diameters from 5 nm to 500 μm. Prior to MIP testing, the bulk samples were pre-dried in the oven at 50 °C for 24 h to remove any moisture inside. The MIP tests were performed in two steps: (i) evacuation of gasses and filling of the penetrometer with mercury in the low-pressure port which increases the pressure up to 50 psi, and (ii) intrusion of mercury into the sample at high-pressure analysis up to 33,000 psi. A  contact angle of 130° and a mercury surface tension of 485 mN/m were used. Intrusion data were collected at an equilibrium rate of 0.001 μL/(g·s) [32].

#### 2.3.5. Indentation

The elasto-plastic properties were measured using nanoindentation tests. These tests were conducted using an Anton Paar (Ashland, VA, USA) nanohardness tester equipped with a Berkovich probe. The principle of nanoindentation is to push a diamond probe against the material under a prescribed vertical force, while measuring the resulting penetration depth. For each sample, an 11 × 11 series of tests was conducted; each test being characterized by a maximum load of 500 mN, a loading/unloading rate of 1000 mN/min, and a holding phase of 10 s. The force resolution was 300 nN and the penetration depth resolution was 0.06 nm, with an acquisition rate of 10.0 Hz. Afterward, for each test, the indentation modulus *M* and indentation hardness *H* were calculated by application of the Oliver and Pharr model [33]:

(2) $$\begin{array}{c} M=\frac{\sqrt{\pi }}{2}\frac{S}{\sqrt{A}};\;H=\frac{P_{max}}{A} \end{array}$$

Here, *S* is the stiffness of the vertical force–penetration depth curve upon unloading, $P_{max}$ is the maximum vertical force, and *A* is the projected contact area at maximum depth. Prior to indentation testing, the projected contact area function was calibrated using fused silica as a reference material.

#### 2.3.6. Scratch Testing

The fracture characteristics were measured using scratch tests conducted using an Anton Paar (Ashland, VA, USA) microscopic scratch tester equipped with a Rockwell probe. The principle of scratch testing is to push a sphero-conical diamond probe across the surface of the material under a linearly increasing vertical force, while measuring the horizontal force and the penetration depth. For each sample, a series of 8 tests was conducted; each test was characterized by a maximum vertical force of 5.5 N, a scratch length of 3 mm, and a scratching speed of 6 mm/min. The horizontal and vertical forces were measured using load sensors with a resolution of 1 mN, and the penetration depth was measured using displacement transducers with a resolution of 3 nm. The fracture toughness was measured for each test by application of nonlinear fracture mechanics [34,35]. Specifically, the fracture toughness $K_{c}$ was computed from the horizontal force $F_{T}$ and penetration depth *d* measurements according to:

(3) $$\begin{array}{c} F_{T}=K_{c}\sqrt{A_{LB}\left(d\right)} \end{array}$$

The horizontally projected load-bearing area is $A_{LB}$ and *p* is the perimeter, both of which are functions of the penetration depth *d*. The probe shape function, $2pA_{LB}$ was calibrated prior to testing using a reference material [36].

## 3. Results

### 3.1. Microstructural Characteristics

**Figure 3:**
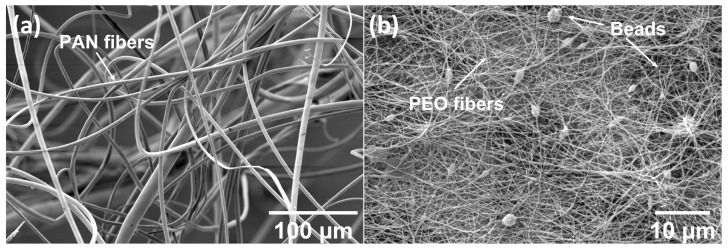
(**a**) Electrospun Polyacrylonitrile (PAN) fibers. (**b**) Electrospun Poly(ethylene oxide) (PEO) fibers.

First, individual fibers are visualized. Figure 3 shows the different morphologies of electrospun PAN and PEO fibers, when collected on a solid metal substrate. The orders of magnitudes of the fiber size are different for both PAN and PEO fibers. The PAN fibers exhibit diameters ranging from 1.68 to 15.23 μm. Meanwhile, the PEO fibers exhibit diameters ranging from 30 to 160 nm. In addition, the surface morphologies are also different. PAN fibers exhibit a smooth surface whereas PEO fibers’ surface show the presence of intermittent beads.

**Figure 4:**
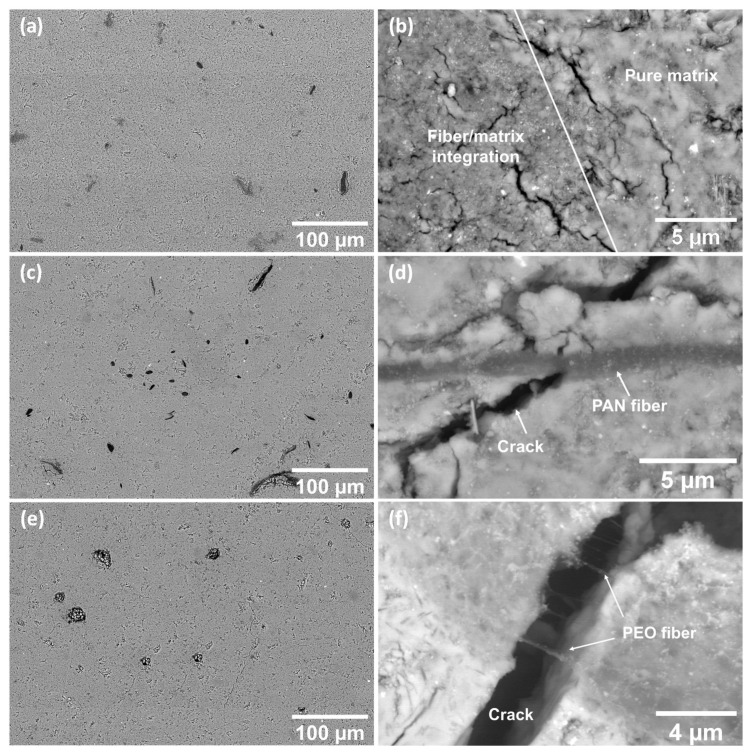
SEM images of electrospun-fiber-reinforced geopolymer composites: (**a**,**b**) KGP-0.5%PAN-W. (**c**,**d**) KGP-0.5%PAN-WG. (**e**,**f**) KGP-0.5%PEO-WG.

Second, fiber-reinforced composites are visualized. Figure 4 displays the SEM images of 0.5 wt% electrospun-fiber-reinforced geopolymer composites. In PAN-fiber-reinforced geopolymer composites (KGP/PAN), electrospun PAN fibers are clearly observed, with a random distribution inside the matrix. For KGP/PAN-W, as shown in Figure 4a, the PAN fibers tend to blend into the matrix and create agglomerations with irregular shapes in the cross section. Figure 4b shows the interface of the fiber agglomeration area and the pure matrix, where the fiber/matrix integration exhibits a different microstructure in terms of color and texture compared to the matrix. For KGP/PAN-WG, as shown in Figure 4c, less fiber agglomeration was observed; instead, individual fibers can be seen with a clear interface with the matrix. The fibers appear to have a cylindrical configuration with a higher aspect ratio. As shown in Figure 4d, the interface between an individual PAN fiber and the matrix shows no porosity, indicating good bonding between the fiber and the matrix. In KGP/PEO-WG, as diplayed in Figure 4e, the microstructure of the composite is significantly different from the KGP/PAN composites. The individual PEO fibers cannot be observed due to the low magnification level of SEM. The PEO beads generated during electrospinning were displayed and appeared to be spherical shapes with geopolymer particles wrapped inside. Individual PEO fibers with nanometer lengthscale can be found in microcracks across the geopolymer matrix and exhibited toughening mechanisms such as crack bridging, as shown in Figure 4f.

Image analysis was performed on the SEM images to achieve a quantification of the fiber morphology inside the geopolymer matrix. The details of the image analysis process can be found in the Supplementary Material and the results are listed in Table 2. For each type of electrospun-fiber-reinforced geopolymer composites, an increasing fiber diameter is observed with increasing fiber weight fraction. KGP/PAN-W exhibited a fiber diameter in the range 3.68–5.08 μm, with the fiber diameter increasing with the weight fraction of PAN. KGP/PAN-WG exhibited a smaller fiber diameter, in the range 3.48–4.57 μm. KGP/PEO-WG displayed the PEO fiber diameter in nanometer lengthscale, which agrees with the PEO fiber shown in Figure 3. The average diameter of PEO fibers within the matrix ranged from 76.7 nm to 105.33 nm. Still, the fiber diameter tends to increase with the reinforcing-polymer weight fraction.

### 3.2. Chemical Characteristics

**Figure 5:**
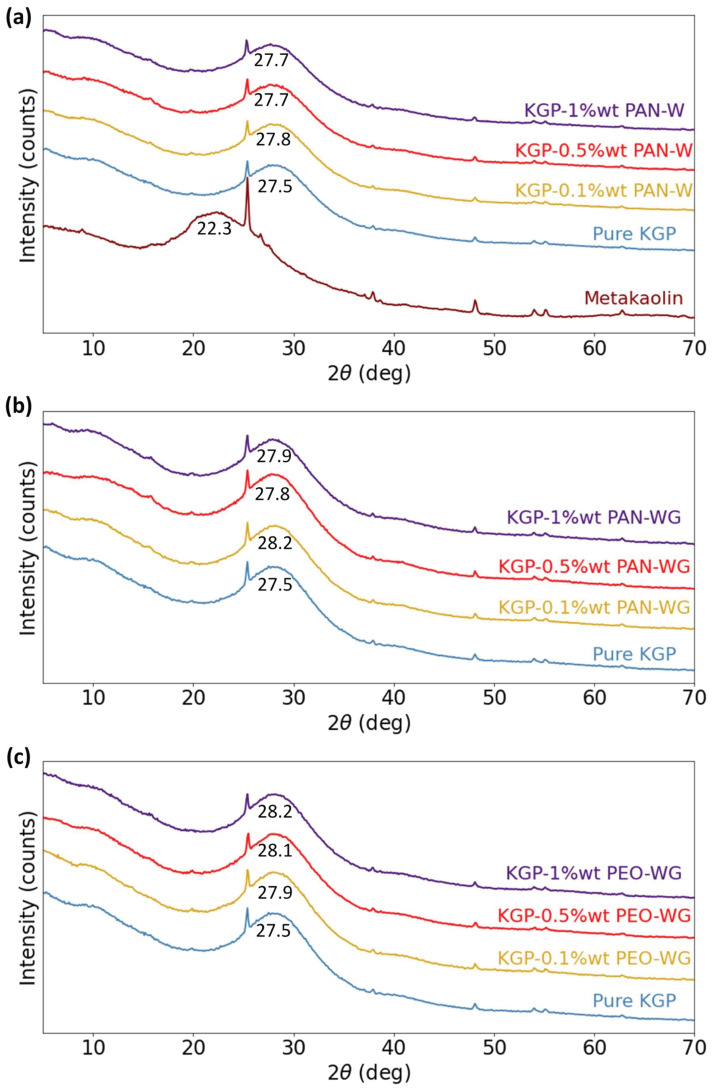
XRD patterns of Metakaolin, KGP and electrospun fiber-reinforced KGP composites: (**a**) Metakaolin, pure KGP and KGP/PAN-W. (**b**) KGP and KGP/PAN-WG. (**c**) KGP and KGP/PEO-WG.

Figure 5a–c shows the XRD patterns for metakaolin, pure geopolymer (KGP), and electrospun-fiber-reinforced KGP composites. It has been established that geopolymers resulting from the polycondensation of various alkali-alumino-silicates are X-ray amorphous [37]. This characteristic is also observed from the XRD patterns of the samples in this study. Metakaolin powder exhibits an amorphous hump centered at 22.3°. It was found that the metakaolin used in this study contained small amounts of anatase impurity, corresponding to the peaks at 25°, 38°, 48°, 54°, 55°, and 63° 2θ. The highly crystalline anatase phase did not participate in the geopolymerization reaction; therefore, anatase can still be observed in the XRD patterns of KGP and KGP-polymer composites. In the XRD pattern of pure KGP, the amorphous hump centered at 27.5° confirmed the formation of geopolymer [26]. For KGP/PAN-W, the hump peaks at 27.8° for 0.1 wt% and 27.7° for 0.5 wt% and 1 wt%. For KGP/PAN-WG, the hump peaks at 28.2° for 0.1 wt%, 27.8° for 0.5 wt% and 27.9° for 1 wt%. For KGP/PEO-WG, the hump peaks at 27.9° for 0.1 wt%, 28.1° for 0.5 wt% and 28.2° for 1 wt%. Compared with pure geopolymer, the peaks slightly shift to larger values of 2θ in the XRD results of all three types of electrospun-fiber-reinforced geopolymer composites, pointing to a change in the lattice size of the geopolymer due to the electrospining process. Among all three types of composites, KGP/PEO-WG exhibits the largest 2θ for the peak, and an increase in the peak location is observed with an increase in fiber-weight ratio. Thus, the electrospinning process affects the molecular structure of the resulting geopolymer nanocomposites.

**Figure 6:**
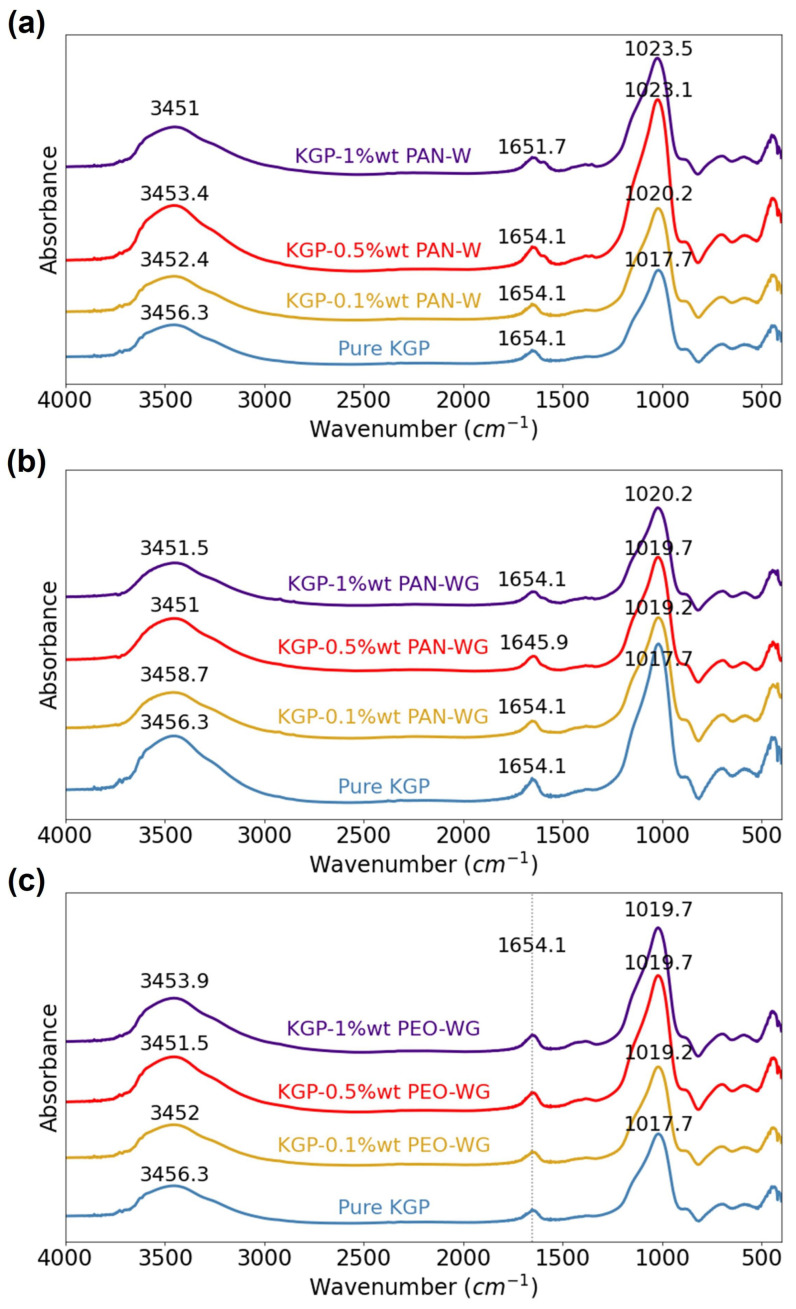
FTIR spectra of KGP and electrospun-fiber-reinforced KGP composites: (**a**) KGP and KGP/PAN-W. (**b**) KGP and KGP/PAN-WG. (**c**) KGP and KGP/PEO-WG.

The FTIR spectra of electrospun-fiber-reinforced geopolymer composites are shown in Figure 6. The bands observed at approximately 3450 cm${}^{-1}$ and 1650 cm${}^{-1}$ in the spectra are commonly assigned to the stretching and bending vibrations of absorbed H${}_{2}$O [38], respectively. The FTIR spectra were analyzed primarily for the “main band” of metakaolin-based geopolymer, which is the broad peak in the region 1200–900 cm${}^{-1}$ and assigned to the asymmetric stretching of Si-O-Si(Al) bonds [39]. For all three types of electrospun-fiber-reinforced geopolymer, the “main band” increases with increasing fiber-weight ratio. In KGP/PAN-W, the peak of the main band slightly shifts from 1017.7 cm${}^{-1}$ to 1023.5 cm${}^{-1}$ with increasing fiber-weight ratio. A slighter increase was also found in KGP/PAN-WG, where the peak location shifts to 1020.2 cm${}^{-1}$ with 1 wt% of PAN fibers, as well as KGP with PEO fibers using waterglass collection. The KGP/PEO-WG composites showed the smallest increase in the peak location, from 1017.7 cm${}^{-1}$ to 1019.7 cm${}^{-1}$. The shift towards a higher wavenumber is related to the shortening of the Si-O-Si(Al) bond, an increase in the bond angle, and, therefore, an increase in the molecular vibrational force constant, indicating a denser structure [40]. Meanwhile, the intensity of the main peak increased in KGP/PAN-W composites and reduced in KGP/PAN-WG composites, which related to the higher amount of the Si-O-Si(Al) bonds sustained in KGP/PAN-W and the lesser quantity of geopolymer formed in KGP/PAN-WG.

### 3.3. 3D Structure Characteristics

**Figure 7:**
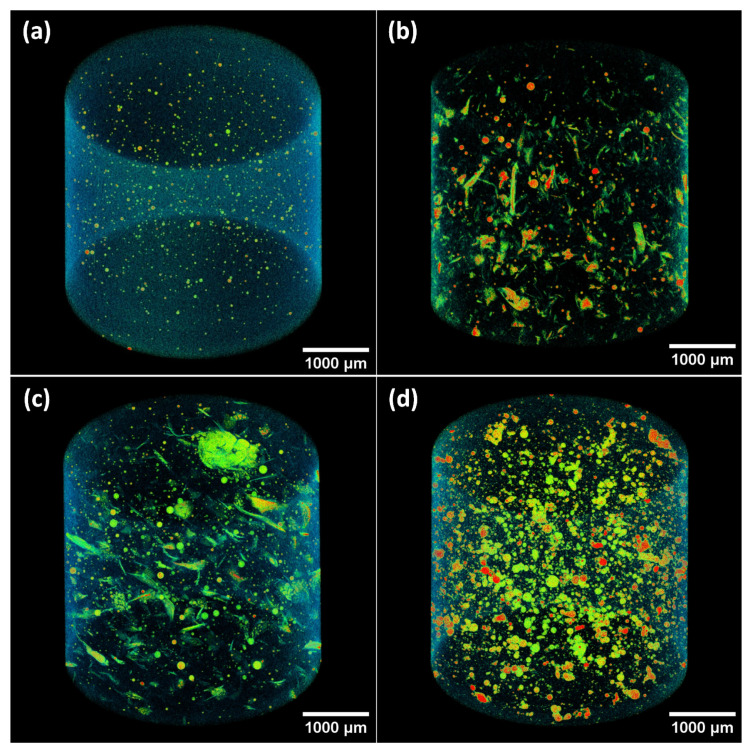
3D Micro-CT reconstruction of electrospun-fiber-reinforced geopolymer composites: (**a**) Pure KGP. (**b**) KGP-0.5%PAN-W. (**c**) KGP-0.5%PAN-WG. (**d**) KGP-0.5%PEO-WG. Geopolymer matrix was filtered and the reddish color indicates existence of air.

The 3D internal microstructure of geopolymer nanocomposites is shown in Figure 7 for the 0.5 wt% electrospun-fiber reinforcement. More Micro-CT results can be found in the Supplementary Materials. Here, the geopolymer matrix has been filtered and only the internal pores and fibers are shown. Due to the resolution of our microCT, 4 μm, individual PAN fibers and PEO beads can be observed, but not individual PEO fibers. The PAN fibers exhibit an elongated fiber configuration whereas the PEO beads appear as sphere-like shapes inside the matrix. These microCT observations agree with the SEM observations, see Figure 4.

The fiber dispersion within the geopolymer matrix is a function of the fiber material and of the liquid-bath collector. PEO electrospun beads were uniformly distributed inside the matrix with a relatively denser distribution compared to PAN fibers. KGP/PAN-W shows an homogeneous dispersion of PAN electrospun fibers, whereas KGP/PAN-WG exhibits fiber clusters. These findings agree with the SEM observations, where PAN fibers tend to blended in the matrix when using water collection. However, they were more challenging to disperse when collected using the waterglass solution.

**Figure 8:**
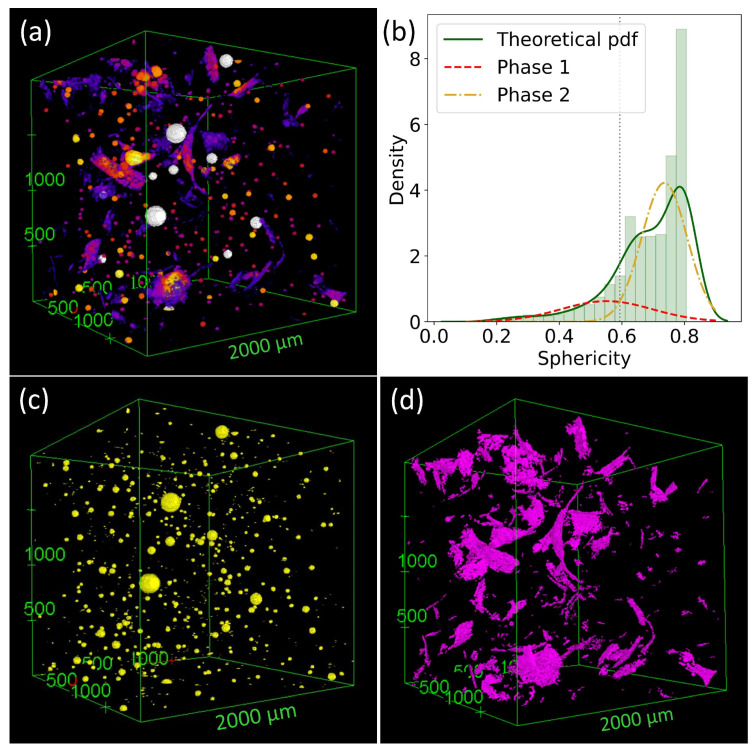
3D Micro-CT image analysis of KGP-0.5%PAN-WG: (**a**) Microstructure. (**b**) Histogram of sphericity of particles. (**c**) Pore structure. (**d**) Fiber structure.

Figure 8a displays the reconstructed microstructure of KGP-0.5%wt PAN-WG, with a gradient color indicating the local thickness of the particles. Figure 8b plots the distribution of the sphericity of all the particles identified and the deconvolution result of the sphericity data. The separated pore structure and the fiber structure are shown in Figure 8c,d, respectively. The analysis was performed on all the electrospun-fiber-reinforced geopolymer composites and the details can be found in the Supplementary Material. After distinguishing pores and fibers, a quantification of the pores and fibers was calculated and is listed in Table 3.

Using microCT, macropores are observed with the average pore size in the range 8.87–29.67 μm. KGP/PAN-W composites showed the smallest average pore diameters among the three types of KGP composites, ranging from 8.87 μm to 15.38 μm. The average pore diameter is in the range of 16.39–19.62 μm for KGP/PAN-WG and 27.17–29.67 μm for KGP/PEO-WG. The pore diameter decreases with increasing fiber fraction in KGP/PAN-W but shows no obvious trend in the other two composites. The porosity calculated is limited to pore sizes larger than 4 μm due to the resolution of Micro-CT. KGP/PAN-W exhibits the smallest macroporosity in the range of 0.1–0.15%. For KGP/PAN-WG, the macroporosity increases from 0.09% to 0.35% with increasing fiber fraction. Similarly, the macropososity increases from 0.1% to 0.31% for KGP/PEO-WG. In terms of fiber structure, the fiber diameters of the PAN fibers appear to be similar to each other, ranging from 10.77–11.53 μm. The larger value compared to the SEM results is due to the resolution limit of the Micro-CT scanning. The well-dispersed and thinner fibers are unable to be identified in Micro-CT, whereas only fibers and fiber clusters with a size greater than 4 μm can be observed and taken into account in the quantification process, causing an overestimation of the fiber diameter. For KGP/PEO-WG, individual PEO fibers are not visualizable due to the limit of Micro-CT resolution (4 μm). The PEO beads are observed and the average size is 29.72–37.02 μm. From the Micro-CT analysis, we are able to achieve a 3D internal structure reconstruction of the electrospun-fiber-reinforced geopolymer composites. Along with SEM, we can develop a basic understanding of the microstructure of the electrospun-fiber-reinforced geopolymer composites.

#### Porosity Measurements

**Figure 9:**
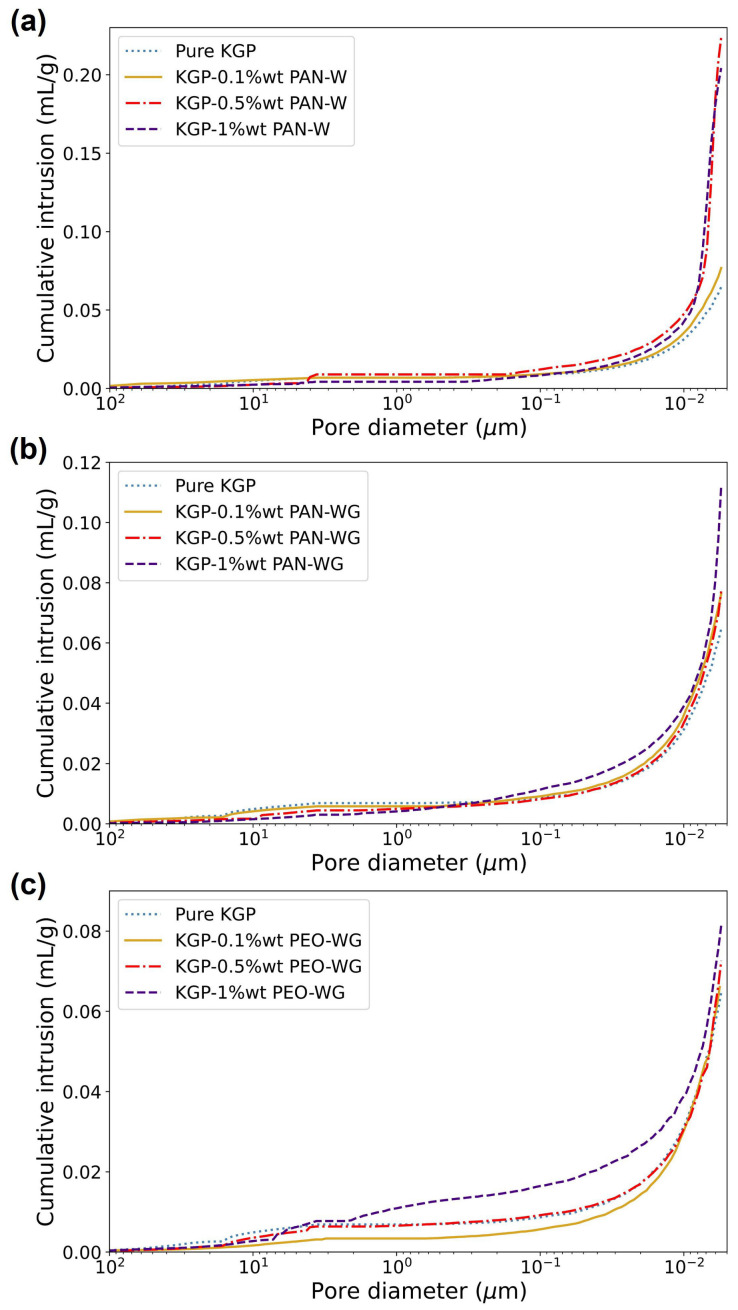
Cumulative MIP results of electrospun-fiber-reinforced geopolymer composites: (**a**) KGP/PAN-W. (**b**) KGP/PAN-WG. (**c**) KGP/PEO-WG.

**Figure 10:**
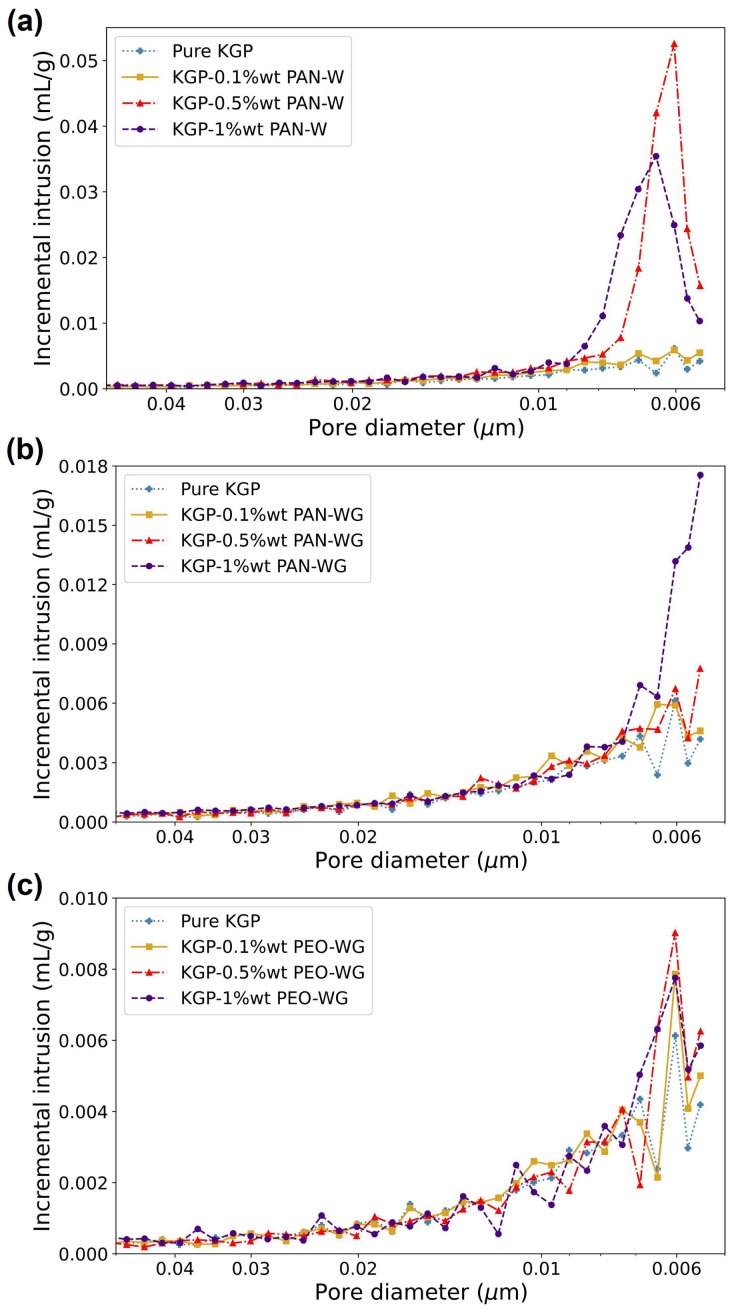
Incremental MIP results of electrospun-fiber-reinforced geopolymer composites: (**a**) KGP/PAN-W. (**b**) KGP/PAN-WG. (**c**) KGP/PEO-WG.

The cumulative intrusion data and incremental intrusion data of eletrospun-fiber-reinforced KGP composites are shown in Figure 9 and Figure 10, respectively. The density obtained from the MIP tests are listed in Table 4. The bulk density is in the range of 1.63–1.96 g/mL for KGP/PAN-W composites, 1.42–1.65 g/mL for KGP/PAN-WG composites and 1.65–2.13 g/mL for KGP/PEO-WG composites. In terms of skeletal density, the range is 1.87–3.51 g/mL for KGP/PAN-W composites, 1.70–1.89 g/mL for KGP/PAN-WG composites and 1.85–2.57 g/mL for KGP/PEO-WG composites, respectively. Compared to pure KGP, the KGP/PAN-WG composites has the smallest bulk density and skeletal density. The KGP/PAN-W composites has a decrease in density in 0.1 wt% fiber fraction, but follows a large increase in density in 0.5 wt% and 1 wt% fiber fraction. The density of KGP/PEO-WG displays a decrease in 0.1 wt% and 0.5 wt% fiber fraction, but an increase in 1.0 wt% fiber fraction.

On the other hand, the porosity results obtained from MIP tests are summarized in Table 5. KGP/PAN-W composites showed a porosity of 12.53% for 0.1 wt% fiber fraction and 37.82–44% for 0.5 wt% and 1.0 wt% fiber fraction. The porosity is significantly increased and resulted in a foam-like material. The porosity of KGP/PAN-WG composites range in 12.58–15.97%, with an increasing trend in higher fiber fractions. The KGP/PEO-WG composites have porosities ranging from 10.90% to 17.44 %, with an increasing value with increased fiber fractions. Moreover, the 0.1 wt% PEO fiber reinforcement decreased the porosity of the composite compared to pure KGP. In general, the electrospun fibers will lead to an increase in the porosity of the geopolymer composite and this can be due to the high open porosity of the electrospun fibers.

From Figure 10, a change in pore diameters caused by the electrospun-fiber reinforcement is also observed. PAN fibers significantly increased the pores with diameters of less than 10 nm and resulted in a decrease in the average pore sizes and mesopore fraction. Compared to pure KGP with an average pore diameter of 10.56 nm, the average pore diameter of KGP-1.0%PAN-W decreased to 8 nm and the associate mesopore fraction increased to 94%. For KGP/PAN-WG, the average pore diameter reduced to 8.7 nm in 1 wt% fiber fraction and the mesoporosity increased to 87%. For KGP/PEO-WG composites, the pore structure was similar to the pure KGP, shown in Figure 10c. Here, the average pore diameter is reduced in low fiber fractions, 0.1 wt% and 0.5 wt%, to 9.24–9.65 nm. However, with 1 wt% PEO fibers, the pore diameter is increased to 10.77 nm and the mesoporosity decreased to 77%. This is due to the increase in macropores introduced in the higher PEO-fiber fractions.

### 3.4. Mechanical Properties

**Figure 11:**
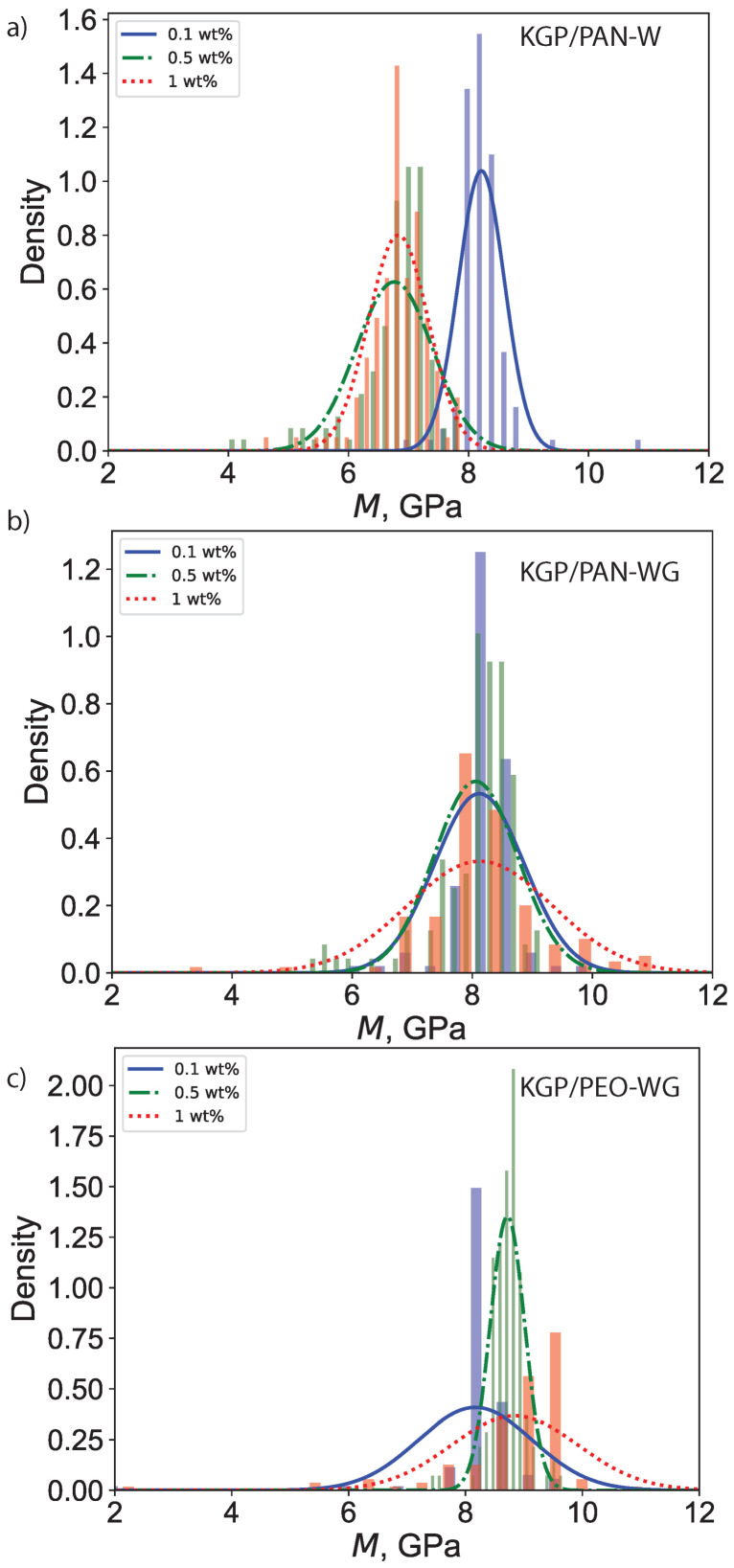
Histogram of indentation modulus *M*: (**a**) KGP/PAN-W. (**b**) KGP/PAN-WG. (**c**) KGP/PEO-WG.

Figure 11 displays the histogram of the indentation modulus for all nine electrospun-fiber-reinforced geopolymer nano-composites and Table 6 lists the average values and standard deviations of the indentation *M* and indentation hardness *H*. The indentation modulus ranges in 6.83–8.29 GPa for KGP/PAN-W, 8.06–8.13 GPa for KGP/PAN-WG, and 8.12–8.90 GPa for KGP/PEO-WG. The indentation hardness ranges in 334.77–401.58 GPa for KGP/PAN-W, 402.61–410.12 GPa for KGP/PAN-WG, and 404.24–452.73 GPa for KGP/PEO-WG. Meanwhile, *M* and *H* decrease with increasing fiber fraction in KGP/PAN-W, but increase with increasing fiber fraction in KGP/PEO-WG. Among the three types of electrospun-fiber-reinforced KGP composites, KGP/PAN-W shows the lowest *M* and *H* while KGP/PEO-WG shows the highest. KGP/PAN-WG displays a similar *M* and *H* with pure KGP, but with a smaller density, as illustrated in the previous section.

The fracture toughness of KGP/PAN-W increased to 0.64 MPa$\sqrt{m}$ in 0.1 wt% but decreased to 0.49 MPa$\sqrt{m}$ for 0.5 wt% and 1.0 wt%. For KGP/PAN-WG, the fracture toughness is reduced for all three fiber-weight ratios. The reduction is higher with increasing fiber-weight ratio. For KGP/PEO-WG, the fracture toughness is increased to the range of 0.69–0.76 MPa$\sqrt{m}$, with a 27% increase observed in KGP-0.5 wt%PEO-WG.

## 4. Discussion

**Figure 12:**
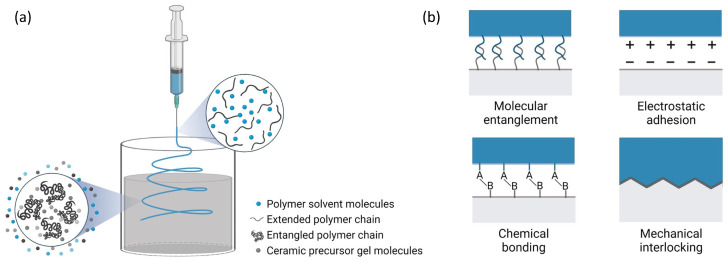
(**a**) The schematic of interactions between the electrospinning polymer solution and the ceramic precursor gel. (**b**) Possible fiber-matrix interfacial bonding mechanisms in the hardened composites.

The interactions among different ingredients in the composite fabrication process is illustrated in Figure 12. When the electrospun fibers are deposited in the ceramic precursor gel, the liquid precursor will facilitate the coagulation of the electrospun fibers through phase exchange between the polymer solvent and the precursor gel [41], as shown in Figure 12a. In nanofiber-reinforced composites, the typical interfacial bonding mechanisms between the nanofibers and the matrix include molecular entanglement, electrostatic adhesion, chemical bonding and mechanical interlocking [42], as presented in Figure 12b. In the hardened geopolymer composites, these mechanisms together are responsible for electrospun-fiber-matrix bonding. On the other hand, due to the multiphysics variables involved in the wet-electrospinning process, the electrospun fibers exhibited great difference in their morphology. From the SEM images of PAN and PEO fibers collected by a solid substrate (Figure 3), the electrospun PAN fibers showed a diameter range of 1.68–15.23 μm and the electrospun PEO fibers displayed a diameter range of 30–160 nm. It has been found that solution properties are the primary factors influencing the diameters of the electrospun fibers. Son et al. [43] explored the relation between an electrospun-PEO-fiber diameter and a solvent and concluded that the higher the dielectric constant of solvent was, the thinner the PEO fiber. The dielectric constant is 80.4 for DI water and 36.7 for DMF, which contributes to the smaller diameter of PEO fibers than the PAN fibers which were dissolved in DMF. Another factor that influences the diameter of the electrospun fibers is the extent of polymer-chain entanglement in the solution. The cyano side-groups of PAN are dipolar in nature and added dipole–dipole attraction to facilitate the entanglement [44]. The much longer chain length and dipolar intermolecular attraction results in much stronger entanglement of PAN compared to PEO. It has also been reported that polymer solutions with poor chain entanglement tend to cause bead formation during electrospinning [45,46]. From SEM results, beads were observed in the PEO solution, which suggests poor polymer chian entanglement due to the low molecular weight of the PEO powder used. The polymer jet tends to split or break up into subjets or droplets if the polymer-chain entanglement is not strong enough. Therefore, in the electrospinning of PEO solutions, subjets were generated and formed PEO fibers with a smaller diameter. Whereas in the electrospinning of PAN fibers, due to the sufficient chain entanglement, a single polymer jet is formed and lands on the substrate to form larger diameter fibers.

From the porosity analysis of the electrospun-fiber-reinforced geopolymer composites, we observe a general trend of an increase in porosity and decrease in average pore size for most of the composites produced. A similar effect of porosity raise by nanofibers has been reported in alumina-based ceramics reinforced with multiwalled carbon nanotubes [47] and aluminum borate nanofiber-reinforced porous ceramics [48]. KGP/PAN-W exhibits a high porosity up to 44%. The high porosity can be related to the combined impact of two effects: alkaline hydrolysis of PAN resulting in water molecules and residual surface porosity of PAN fibers after solvent evaporation. Moreover, a pore size refinement effect was observed. The average pore size was reduced to 7.7 nm with a concentration range within 5 nm. The high porosity as well as narrow range of pore-size distribution are desired in micro- and nanomembranes [49,50] and ceramic filtration systems [51] and provide great potential in environmental remediation applications. Traditional electrospun nanofiber mats have been extensively researched in air filtration and water filtration because of their high open porosity and adjustable porous structure [52,53,54,55]. Multistructured electrospun fiber membranes have been proven to be remarkably effective in air filtration [54,56,57]. Li et al. [58] controllably fabricated beaded fiber structure via electrospinning to reduce the pressure drop and increase the air filtering efficiency. Zhang et al. [59] manufactured tree-like porous cellulose nanofiber membranes with large surface areas up to 19.8 m^2^/g, leading to a highly efficient heavy-metal adsorbent. From the MIP analysis of our study, the cumulative pore area can go as high as 116.43 m^2^/g in KGP-0.5%PAN-W, providing substantial opportunities in waste treatment. Moreover, despite the various capabilities of electrospun fiber mats, pure electrospun nanofibers pose major challenge in industrial production. A 1 g weighted electrospun nanofibrous mat typically takes hours to form. Therefore, the proposed electrospun-fiber-reinforced geopolymer composites with an increase in porosity, reduced average pore size, and increased weight efficiency can lead to applications such as scalable water and air filtration systems.

In KGP/PAN-W, the PAN fibers enter into the geopolymerization reaction from the first stage. Here, raw materials including SiO${}_{2}$ and KOH both interact with the PAN fibers, resulting in an increase in the surface area and mesoporosity of PAN fibers [60]. The high surface area of PAN fibers in reverse facilitates the geopolymer formation, leading to a denser material. However, the densification of the material was mitigated by the increasing porosity generated, which caused the decrease in mechanical properties in KGP/PAN-W. The mechanical strength of KGP/PAN-WG were similar compared to pure geopolymer, but the material displays a smaller density and finer pore structure. The KGP/PEO-WG displayed an increase in mechanical strength due to the nanoscale reinforcement of PEO fibers. On the other hand, the fracture toughness of KGP/PAN-W showed an increase in 0.1 wt% fiber fraction. However, the toughening effect is counteracted by the increase in porosity in higher weight fractions. The fracture toughness of KGP/PEO-WG was enhanced up to 27% in 0.5 wt% fiber fraction. The mechanisms for toughening the material were identified as a crack-bridging effect. With higher fiber ratios, the enhancement may be mitigated by the fiber agglomerations occurring. The wet-electrospun fibers provide great potential in generating lightweight materials with desired mechanical properties and a toughened yet porous material. This novel material provides tremendous opportunities in engineering applications such as construction materials, automotive parts, structural components, wear-resistant materials, etc. In particular, the advantages of lightweight and high performance are of prime interest in aerospace structures, such as equipment enclosures [61], aircraft interiors, coatings [62], crew gear [63], solar array substrates [64,65], etc. Therefore, our proposed electrospun-fiber-reinforced ceramic composites shows great potential in future design and manufacturing of aerospace, automotive and civil structural components.

## 5. Conclusions

This study investigated the influence of liquid collector and polymer on the chemistry, microstructure, and mechanical properties of fiber-reinforced geopolymers synthesized by depositing wet-electrospun polymeric fibers within a geopolymer precursor solution. Here are our main results:

- The wet-electrospinning process preserves the amorphous structure of the resulting geopolymer composites but leads to changes in the molecular structure. XRD results show a change in the location of the hump and FTIR results show an increase in the position of the main band of geopolymer for increasing fiber-weight ratio.
- The diameter of the electrospun fiber is dictated by the chemistry of the polymer: PAN yields micron-sized fibers with fiber diameters in the range 1.68–15.23 μm whereas PEO yields nano-sized fibers with fiber diameters in the range 30–160 nm.
- For low fiber-weight ratios, 0.1 wt%, the wet-electrospinning process leads to a 10% reduction in both the bulk density and the skeletal density. In addition, an increase in both the mesoporosity and the overall porosity is observed.
- The mechanical properties of wet-electrospun-fiber-reinforced geopolymers depend on both the polymer type and the liquid collector. For KGP/PAN-W, both the stiffness and fracture toughness decrease with increasing fiber-weight ratio; for KGP/PAN-WG, steady values are observed; for KGP/PEO, both the indentation modulus and fracture toughness increase with the presence of wet-electrospun fibers.

These results demonstrate the viability of wet electrospinning inside a geopolymer precursor gel to yield light nanofiber-reinforced ceramics. Our next step will be investigating the thermal characteristics of the electrospun-fiber-reinforced geopolymer composites and further enhancement of the mechanical performance.

## Figures and Tables

**Table 1:** Mix design for the geopolymer nanocomposites reinforced with electrospun polyacrylonitrile (PAN) and poly(ethylene oxide) (PEO), that were synthesized in this study.

Sample Name	Polymer	Liquid Bath Collector	Fiber Weight Ratio
KGP-0.1%PAN-W	PAN	DI Water	0.1 wt%
KGP-0.5%PAN-W	PAN	DI Water	0.5 wt%
KGP-1.0%PAN-W	PAN	DI Water	1 wt%
KGP-0.1%PAN-WG	PAN	Waterglass	0.1 wt%
KGP-0.5%PAN-WG	PAN	Waterglass	0.5 wt%
KGP-1.0%PAN-WG	PAN	Waterglass	1 wt%
KGP-0.1%PEO-WG	PEO	Waterglass	0.1 wt%
KGP-0.5%PEO-WG	PEO	Waterglass	0.5 wt%
KGP-1.0%PEO-WG	PEO	Waterglass	1 wt%

**Table 2:** SEM image analysis results of Polyacrylonitrile (PAN) and Poly(ethylene oxide) (PEO) electrospun fibers inside geopolymer composites.

Sample	Fiber Weigh Ratio	Fiber Diameter (nm)
KGP-0.1%PAN-W	0.1 wt%	3.68 × 10${}^{3}$ ± 1.81 × 10${}^{3}$
KGP-0.5%PAN-W	0.5 wt%	5.67 × 10${}^{3}$ ± 3.44 × 10${}^{3}$
KGP-1.0%PAN-W	1.0 wt%	5.08 × 10${}^{3}$ ± 3.48 × 10${}^{3}$
KGP-0.1%PAN-WG	0.1 wt%	3.48 × 10${}^{3}$ ± 1.73 × 10${}^{3}$
KGP-0.5%PAN-WG	0.5 wt%	4.40 × 10${}^{3}$ ± 3.19 × 10${}^{3}$
KGP-1.0%PAN-WG	1.0 wt%	4.57 × 10${}^{3}$ ± 4.30 × 10${}^{3}$
KGP-0.1%PEO-WG	0.1 wt%	90.58±37.37
KGP-0.5%PEO-WG	0.5 wt%	76.60±42.58
KGP-1.0%PEO-WG	1.0 wt%	105.33±52.24

**Table 3:** Micro-CT Image Analysis Results of Polyacrylonitrile (PAN) and Poly(ethylene oxide) (PEO) electrospun fibers inside geopolymer composites.

Sample	Pore Diameter (μm)	Porosity	Fiber Diameter (μm)	Fiber Volume Fraction	Fiber Surface Area (m^2^/g)
Pure KGP	15.24±11.54	0.07%	0	0%	0
KGP-0.1%PAN-W	15.38	0.12%	11.26	0.11%	0.18
KGP-0.5%PAN-W	9.30	0.10%	11.04	0.68%	0.15
KGP-1.0%PAN-W	8.87	0.15%	10.77	2.11%	0.27
KGP-0.1%PAN-WG	19.62	0.09%	11.13	0.12%	0.17
KGP-0.5%PAN-WG	16.39	0.17%	10.93	0.68%	0.18
KGP-1.0%PAN-WG	19.07	0.35%	11.53	2.39%	0.19
KGP-0.1%PEO-WG	28.38	0.10%	–	0.11%	0.18
KGP-0.5%PEO-WG	27.17	0.19%	–	0.88%	0.23
KGP-1.0%PEO-WG	29.67	0.31%	–	2.65%	0.29

**Table 4:** Density of electrospun-fiber-reinforced geopolymer composites from MIP tests.

Sample	Bulk Density (g/mL)	Skeletal Density (g/mL)
Pure KGP	1.78	2.02
KGP-0.1%PAN-W	1.63	1.87
KGP-0.5%PAN-W	1.96	3.51
KGP-1.0%PAN-W	1.85	2.98
KGP-0.1%PAN-WG	1.65	1.89
KGP-0.5%PAN-WG	1.61	1.84
KGP-1.0%PAN-WG	1.42	1.70
KGP-0.1%PEO-WG	1.65	1.85
KGP-0.5%PEO-WG	1.66	1.89
KGP-1.0%PEO-WG	2.13	2.57

**Table 5:** Porosity results of electrospun-fiber-reinforced geopolymer composites from MIP tests.

Sample	Porosity	Mesopore Fraction	Avg Pore Diameter (nm)
Pure KGP	11.55%	84%	10.56
KGP-0.1%wtPAN-W	12.53%	86%	10.20
KGP-0.5%wtPAN-W	44.00%	93%	7.70
KGP-1.0%wtPAN-W	37.82%	94%	8.00
KGP-0.1%wtPAN-WG	12.58%	85%	10.33
KGP-0.5%wtPAN-WG	12.37%	87%	9.77
KGP-1.0%wtPAN-WG	15.97%	87%	8.7
KGP-0.1%wtPEO-WG	10.90%	89%	9.24
KGP-0.5%wtPEO-WG	12.07%	85%	9.65
KGP-1.0%wtPEO-WG	17.33%	77%	10.77

**Table 6:** Mechanical properties of electrospun-fiber-reinforced geopolymer composites. *M* is the average indentation modulus, *H* is the average indentation hardness, and $K_{c}$ is the average fracture toughness.

Sample	*M* (GPa)	*H* (GPa)	$K_{c}$ (MPa$\sqrt{m}$)
Pure Geopolymer	8.30 ± 0.25	430.43 ± 23.34	0.60 ± 0.02
KGP-0.1%PAN-W	8.29 ± 0.39	402.58 ± 38.85	0.64 ± 0.02
KGP-0.5%PAN-W	6.76 ± 0.64	334.77 ± 53.10	0.49 ± 0.01
KGP-1.0%PAN-W	6.83 ± 0.50	347.10 ± 50.90	0.49 ± 0.02
KGP-0.1%PAN-WG	8.12 ± 0.75	410.12 ± 54.68	0.57 ± 0.01
KGP-0.5%PAN-WG	8.06 ± 0.70	403.50 ± 56.22	0.53 ± 0.03
KGP-1.0%PAN-WG	8.13 ± 1.21	402.61 ± 70.91	0.49 ± 0.01
KGP-0.1%PEO-WG	8.12 ± 0.90	404.24 ± 63.27	0.69 ± 0.01
KGP-0.5%PEO-WG	8.72 ± 0.30	433.26 ± 28.70	0.76 ± 0.01
KGP-1.0%PEO-WG	8.90 ± 0.90	452.73 ± 59.04	0.69 ± 0.04

## Data Availability

Data is contained within the article or Supplementary Material.

## Supplementary Materials

The following supporting information can be downloaded at: https://www.mdpi.com/article/10.3390/polym14193943/s1.

## Abbreviations

The following abbreviations are used in this manuscript:

PAN	Polyacrylonitrile
PEO	Poly(ethylene oxide)
KGP	Potassium geopolymer
SEM	Scanning Electron Microscopy
XRD	X-ray Diffraction
FTIR	Fourier Transform Infrared Spectroscopy
MIP	Mercury Intrusion Porosimetry
Micro-CT	X-ray Micro-Computed Tomography
