# Supplementary material for: Novel Wet Electrospinning Inside a Reactive Pre-Ceramic Gel to Yield Advanced Nanofiber-Reinforced Geopolymer Composites
Source: Polymers (Basel). 2022 Sep 21;14(19):3943. doi: 10.3390/polym14193943 (PMC9572844; doi:10.3390/polym14193943)
Supplement: Supplementary file 1 [file polymers-14-03943-s001.zip › polymers-1875660-supplementary.pdf]

Supplementary Material

Yunzhi Xu[1], Ping Guo[2,*] Ange-Therese Akono[1,2,*]
[1] Department of Civil and Environmental Engineering, Northwestern University, Evanson, IL, USA
[2] Department of Mechanical Engineering, Northwestern University, Evanson, IL, USA
*Corresponding authors: Ange-Therese Akono: ange-therese.akono@northwestern.edu
Ping Guo ping.guo@northwestern.edu

1. Scanning Electron Microscopy Images

This section displays the microstructure of all 9 electrospun fiber reinforced geopolymer composites.

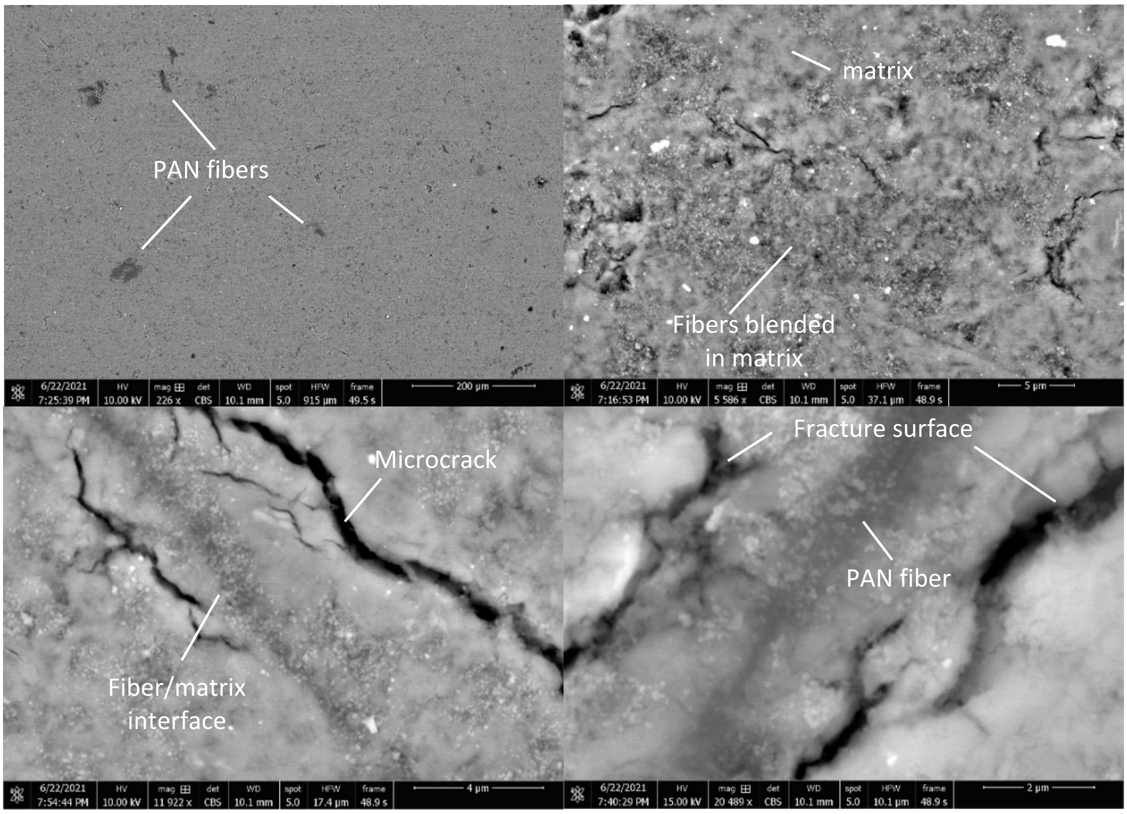

Figure S1: Microstructure of KGP-0.1%wt PAN-W.

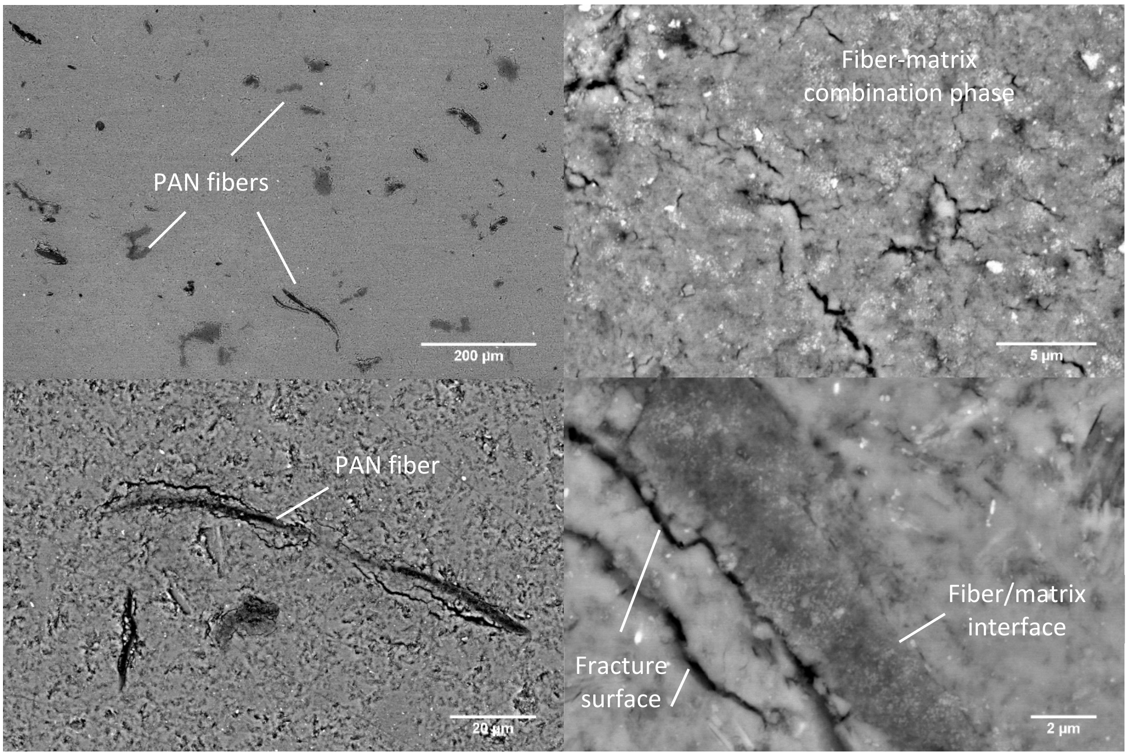

Figure S2: Microstructure of KGP-0.5%wt PAN-W.

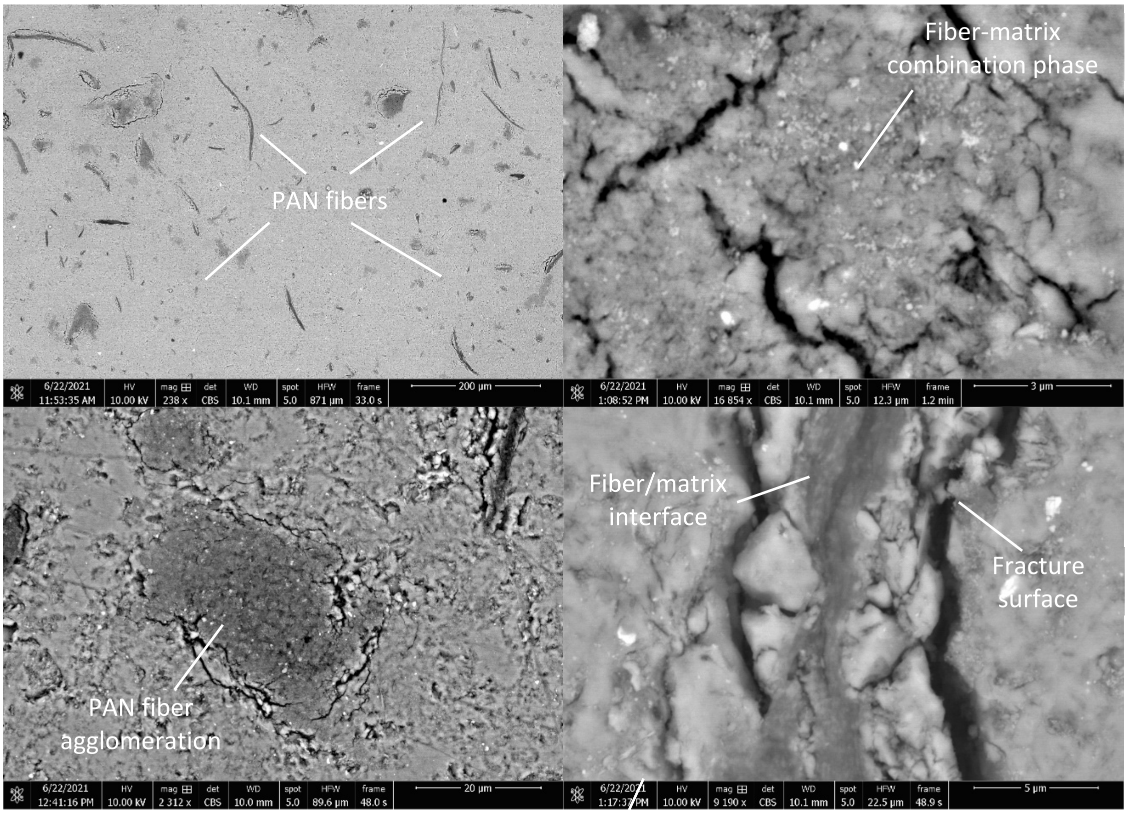

Figure S3: Microstructure of KGP-1.0%wt PAN-W.

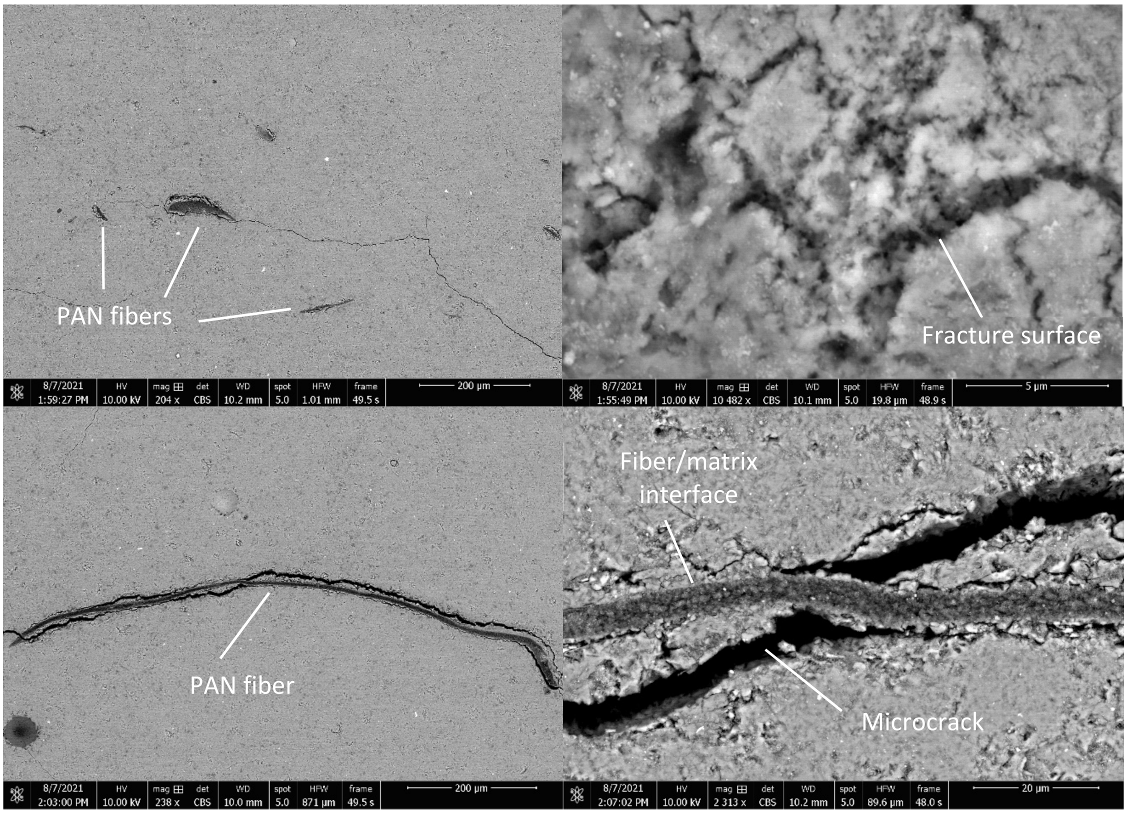

Figure S4: Microstructure of KGP-0.1%wt PAN-WG.

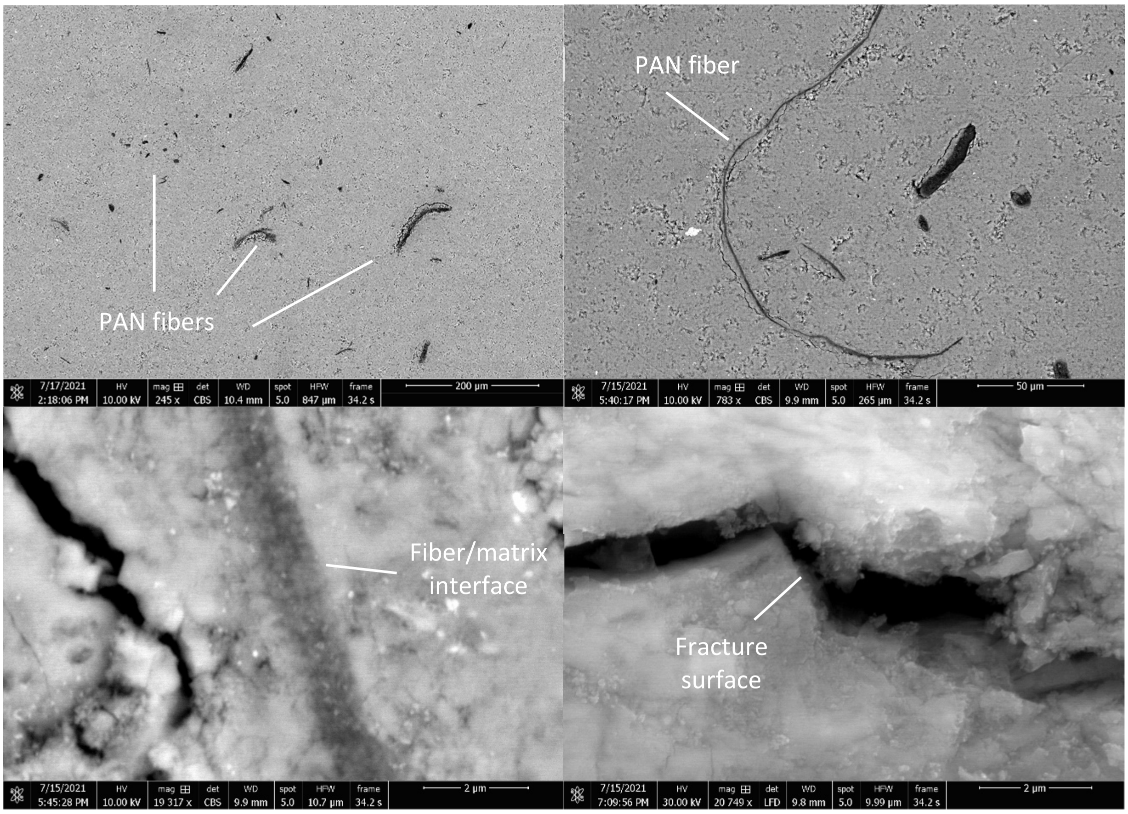

Figure S5: Microstructure of KGP-0.5%wt PAN-WG.

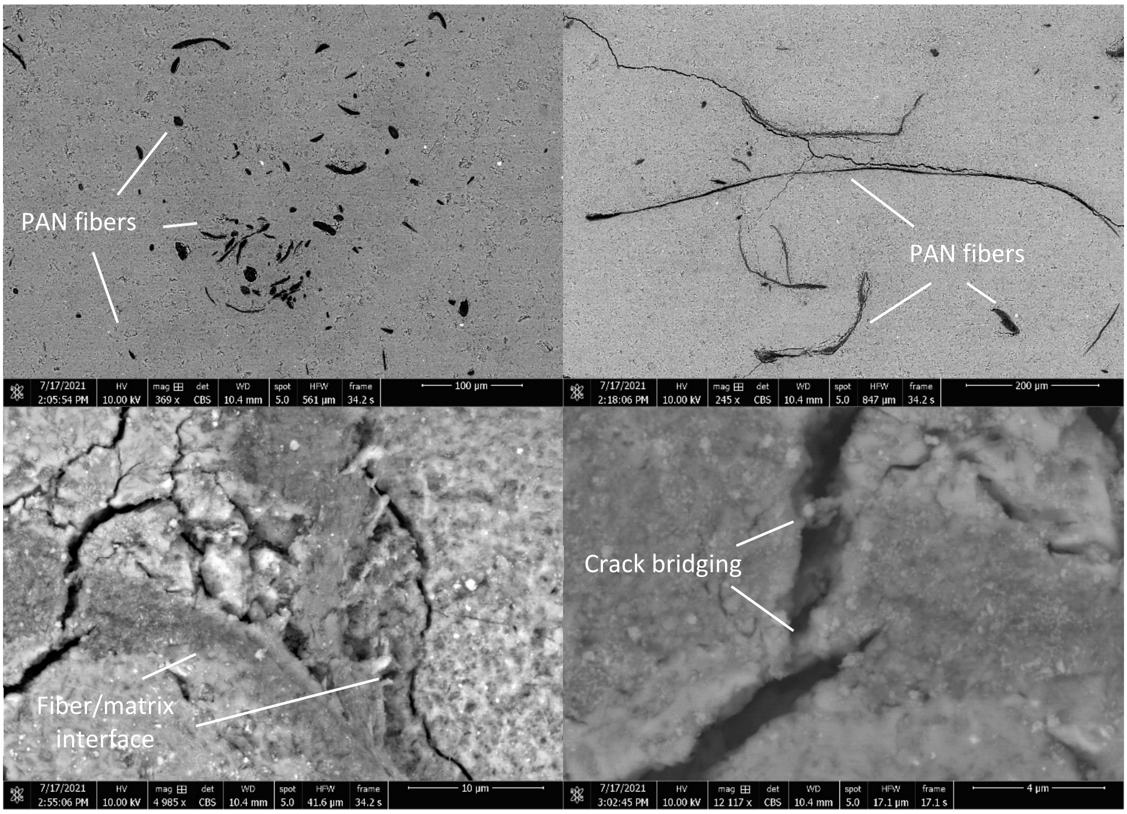

Figure S6: Microstructure of KGP-1.0%wt PAN-WG.

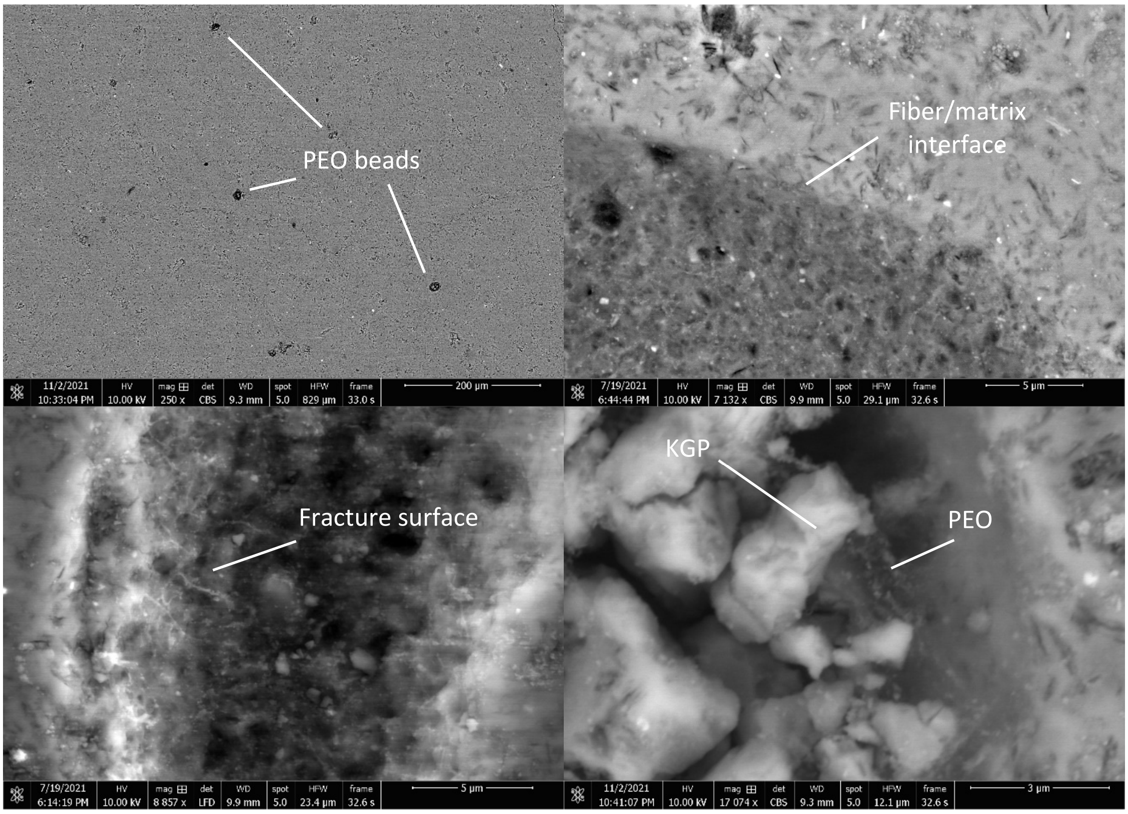

Figure S7: Microstructure of KGP-0.1%wt PEO-WG.

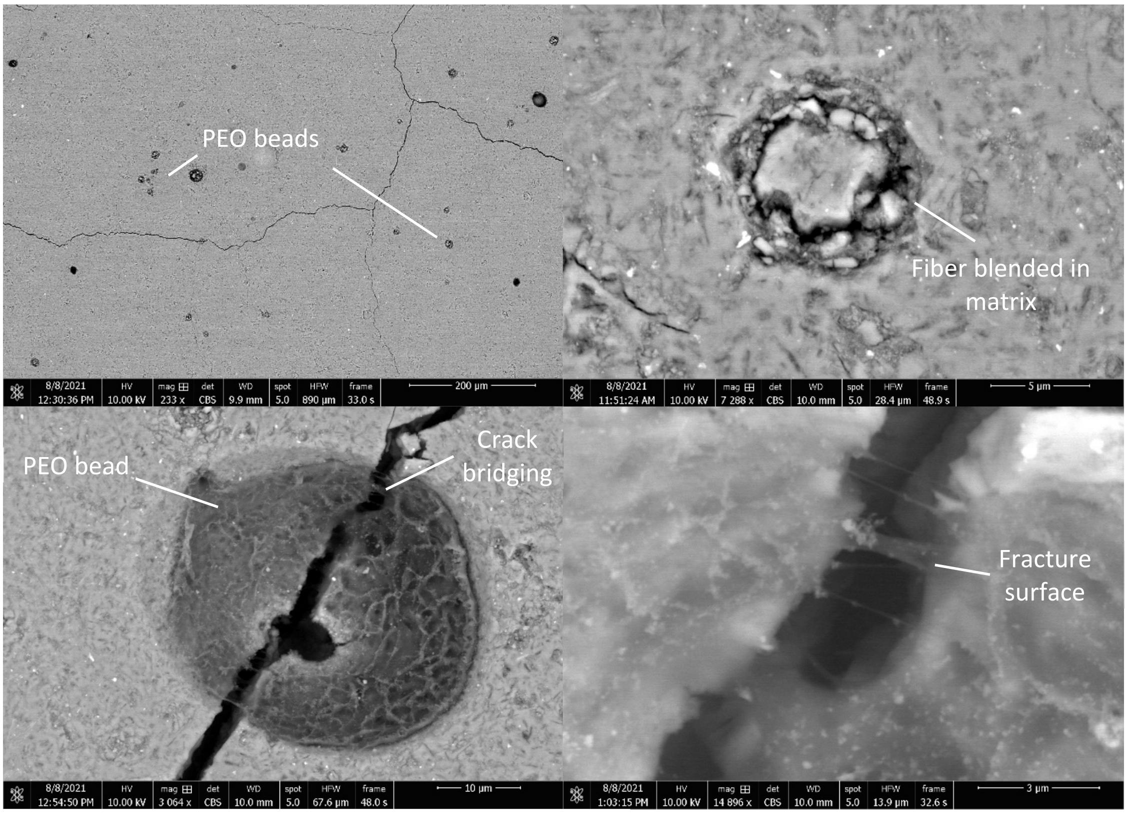

Figure S8: Microstructure of KGP-0.5%wt PEO-WG.

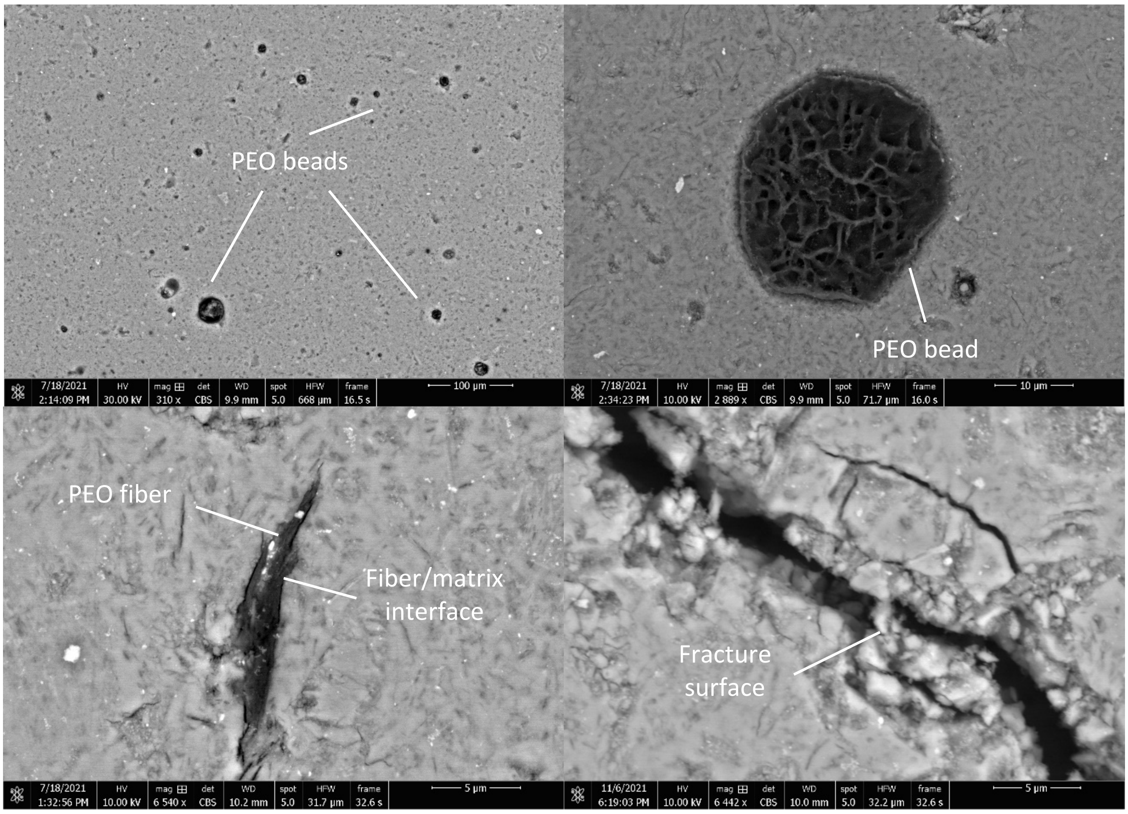

Figure S9: Microstructure of KGP-1.0%wt PEO-WG.

2. Image Analysis on SEM Images

Figure S10 shows the procedure of image analysis based on the SEM images of hardened geopolymer composites for calculation of the diameters of electrospun fibers. For each sample, the SEM images were taken in a 10 x 10 grid with a magnification of 1000 x, spanning a total area of 2072 µm x 1381 µm, shown in Figure S10(a). The histogram of the greyscale values were plotted and a deconvolution process based on the variational Bayesian Gaussian mixture model was performed on the grayscale histogram to obtain the threshold for image segmentation, shown in Figure S10(b). Figure S10(c) displays the binary image after image segmentation and Figure S10(d) shows the labeled image where each fiber is labeled uniquely. The diameter of the fibers are calculated using ImageJ and Figure S10(e) displays the thickness map of the fibers.

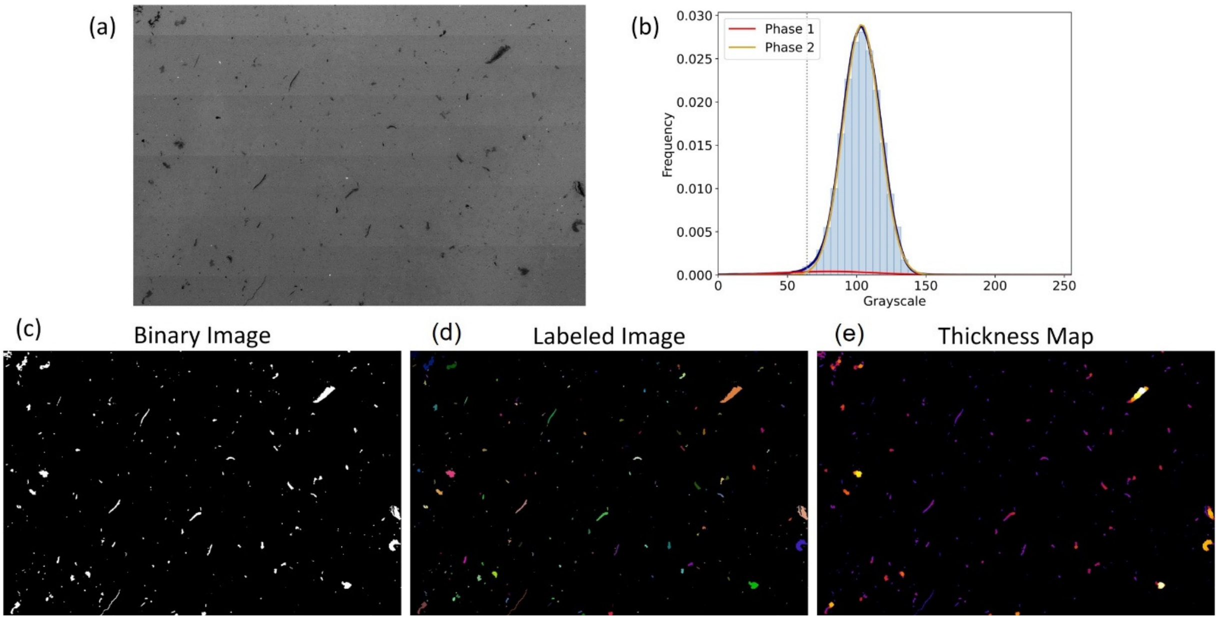

Figure S10: Image analysis procedure of SEM images. (a) Original SEM image; (b) Deconvolution of grayscale histogram; (c) Identification of individual fibers and the thickness(diameter) map.

3. Micro-CT Image Reconstruction

This section displays the 3D microstructural reconstruction of 9 electrospun fiber reinforced geopolymer composites based on the Micro-CT scannings.

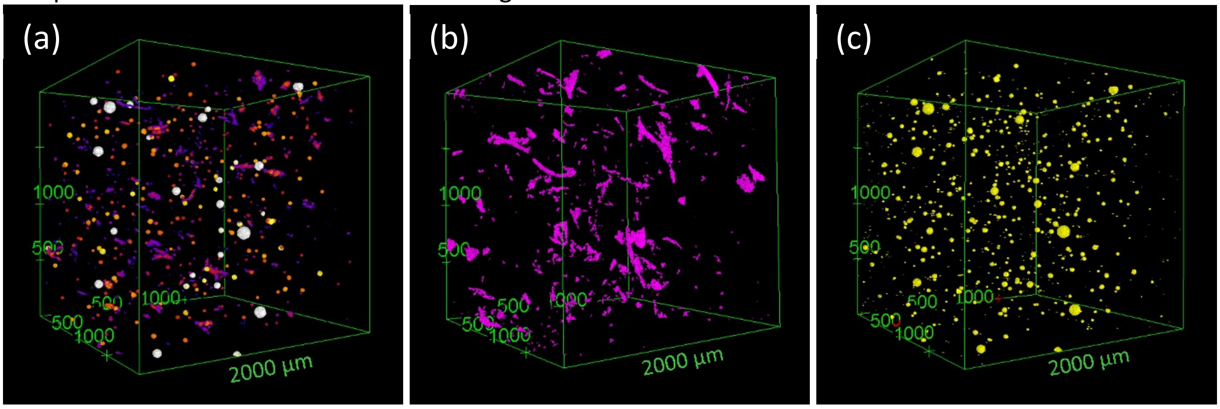

Figure S11: 3D reconstruction of KGP-0.1%wt PAN-W. (a) 3D microstructure; (b) PAN fiber distribution; (c) Pore distribution.

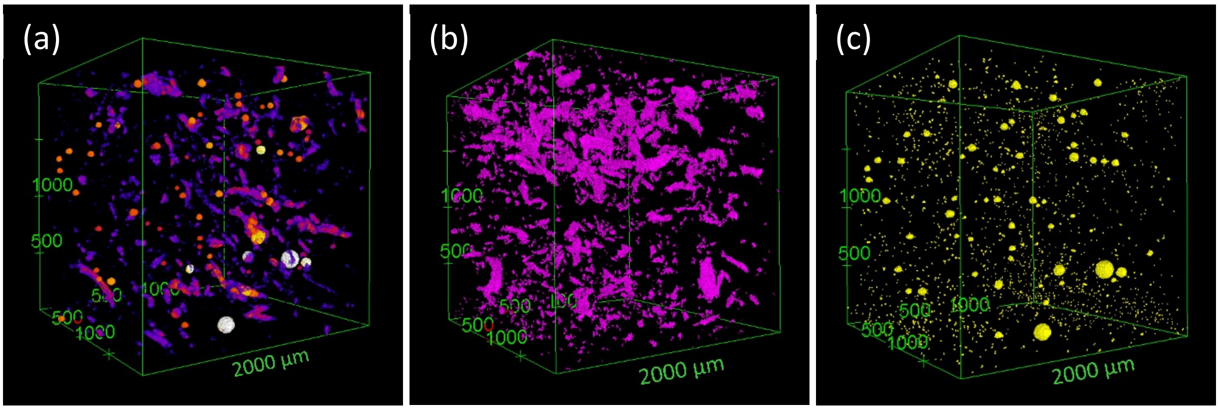

Figure S12: 3D reconstruction of KGP-0.5%wt PAN-W. (a) 3D microstructure; (b) PAN fiber distribution; (c) Pore distribution.

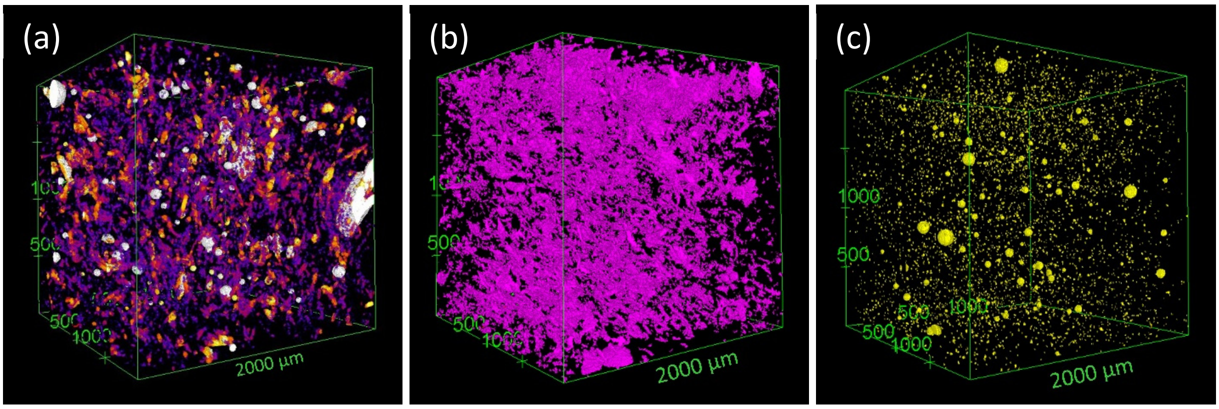

Figure S13: 3D reconstruction of KGP-1.0%wt PAN-W. (a) 3D microstructure; (b) PAN fiber distribution; (c) Pore distribution.

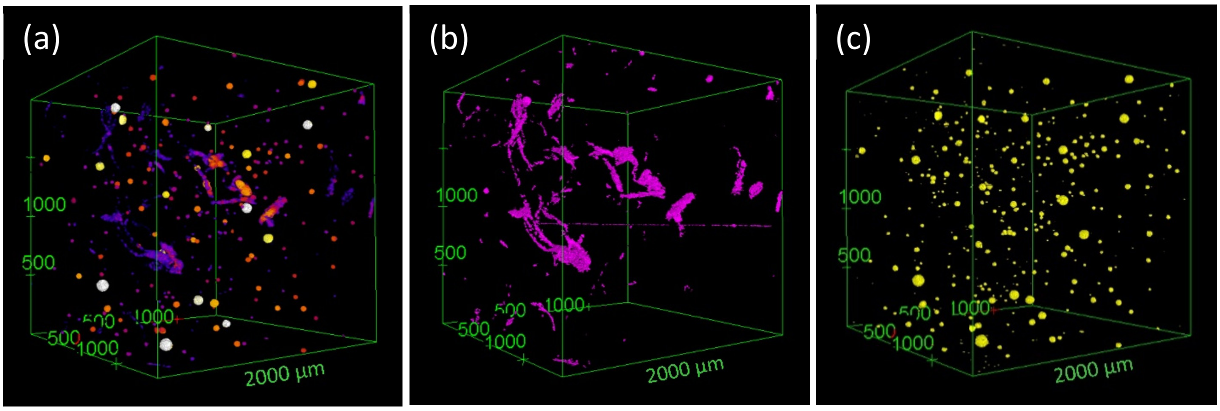

Figure S14: 3D reconstruction of KGP-0.1%wt PAN-WG. (a) 3D microstructure; (b) PAN fiber distribution; (c) Pore distribution.

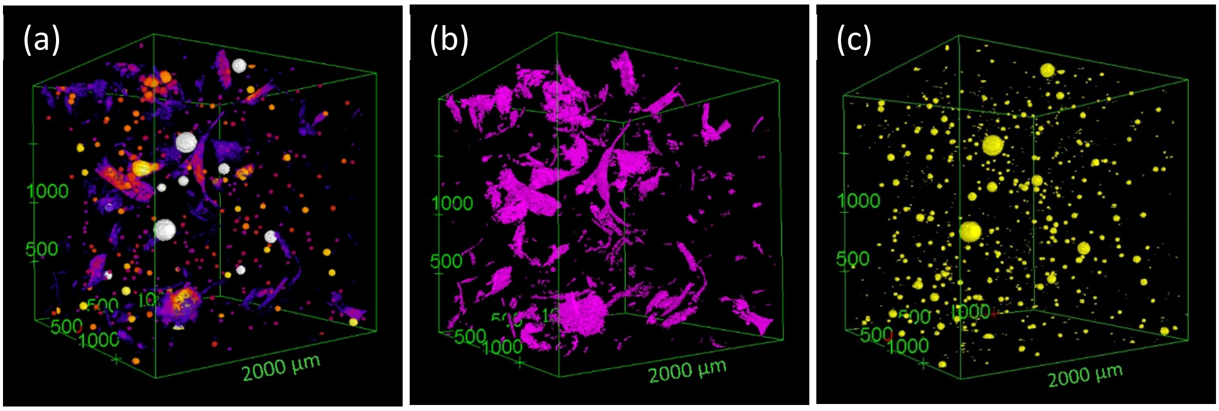

Figure S15: 3D reconstruction of KGP-0.5%wt PAN-WG. (a) 3D microstructure; (b) PAN fiber distribution; (c) Pore distribution.

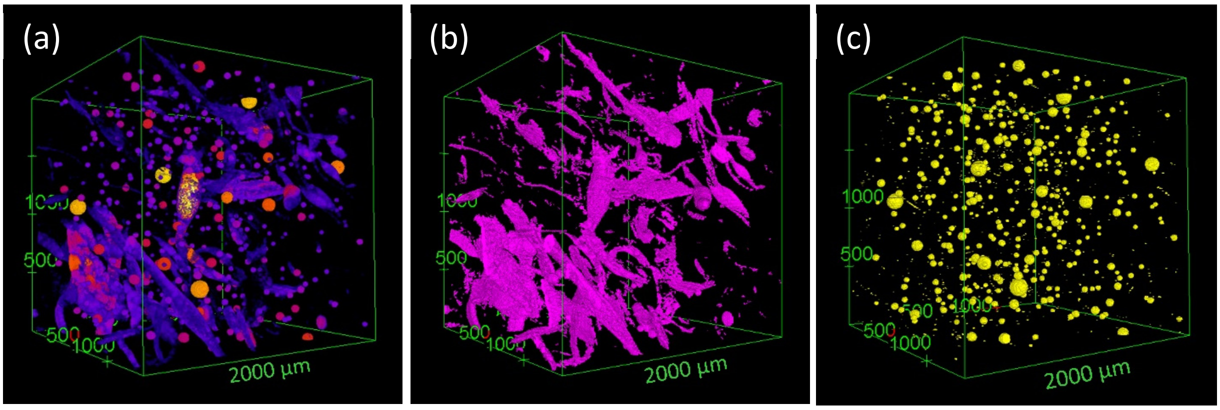

Figure S16: 3D reconstruction of KGP-1.0%wt PAN-WG. (a) 3D microstructure; (b) PAN fiber distribution; (c) Pore distribution.

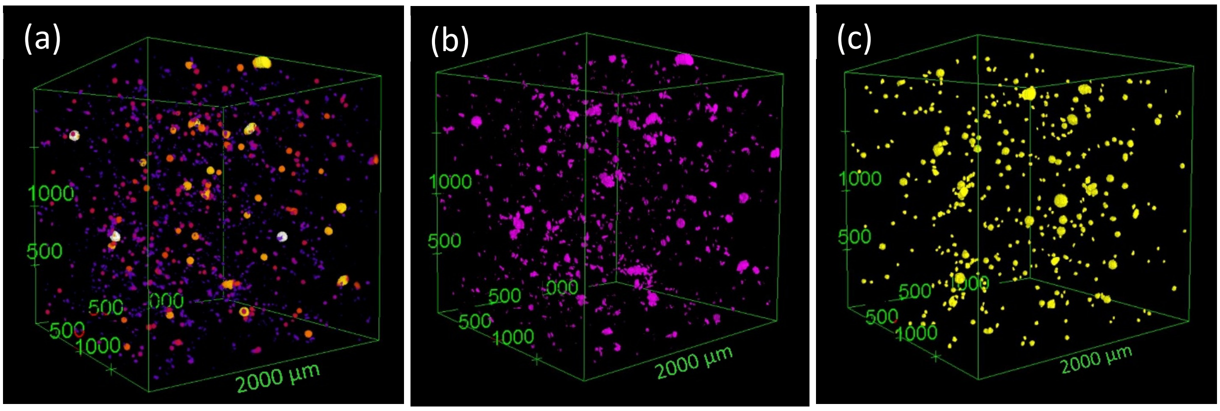

Figure S17: 3D reconstruction of KGP-0.1%wt PEO-WG. (a) 3D microstructure; (b) PAN fiber distribution; (c) Pore distribution.

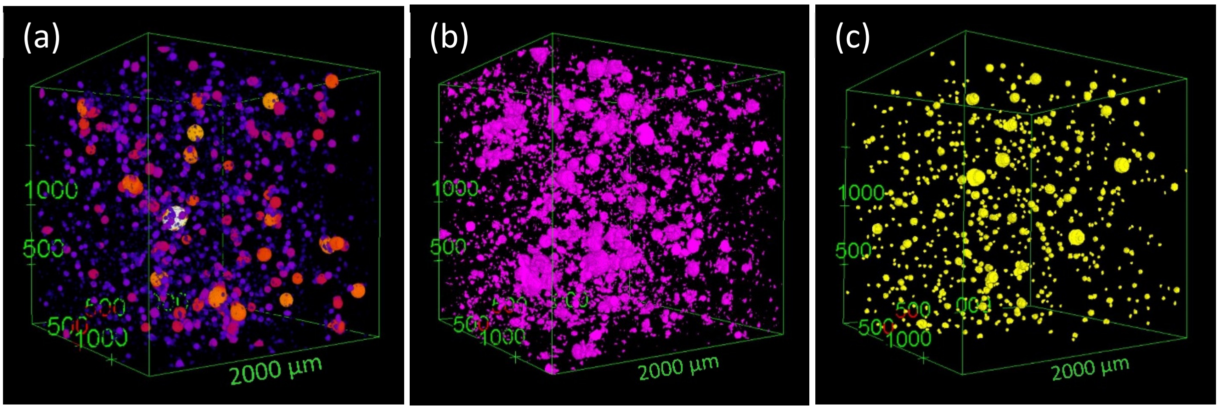

Figure S18: 3D reconstruction of KGP-0.5%wt PEO-WG. (a) 3D microstructure; (b) PAN fiber distribution; (c) Pore distribution.

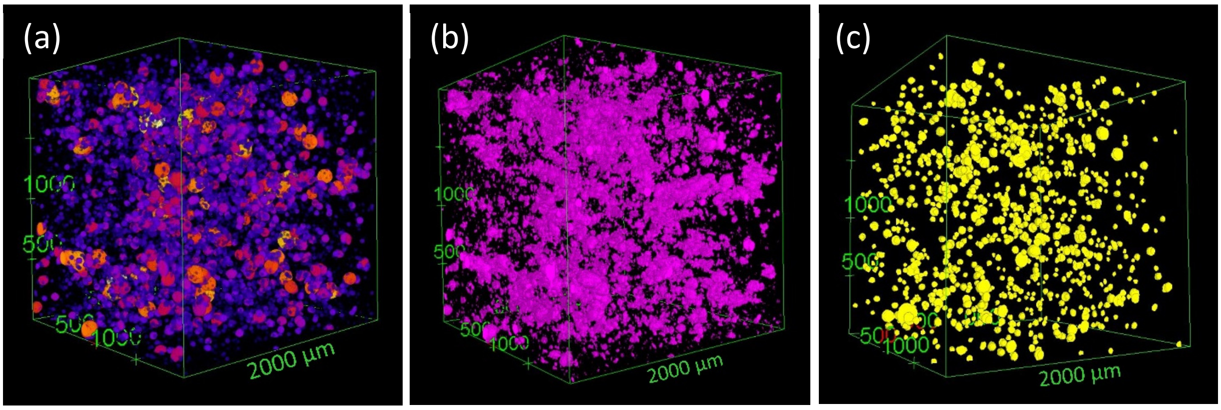

Figure S19: 3D reconstruction of KGP-1.0%wt PEO-WG. (a) 3D microstructure; (b) PAN fiber distribution; (c) Pore distribution.

4. Indentation Test Results

Figure S20 displays the histograms of the indentation hardness for all nine geopolymer nanocomposites.

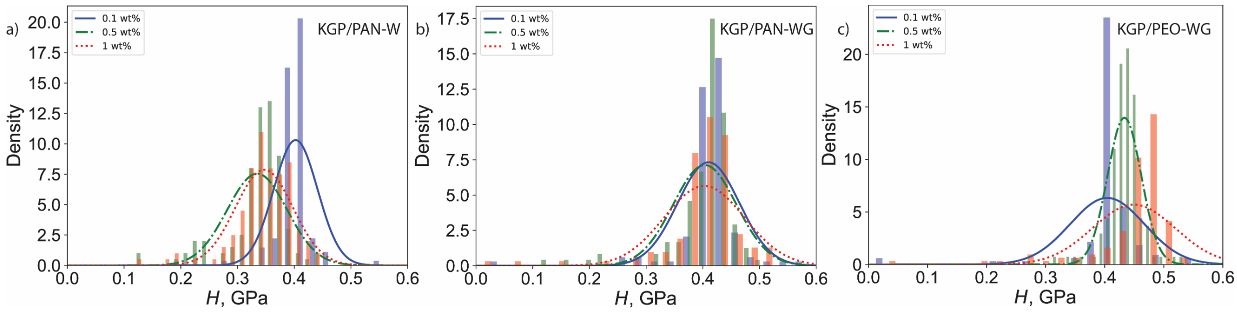

Figure S20: Histogram of indentation hardness H for electrospun-fiber geopolymer composites.

5. Scratch Test Results

Figures S21-S23 display the scaling of the horizontal force F_T during scratch tests.

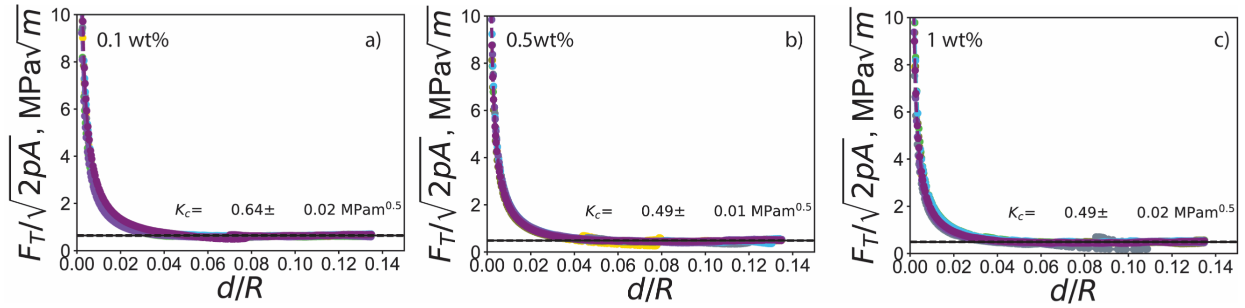

Figure S21: Scaling of scratch force for for KGP_PAN_Water. $F_T$is the horizontal force, p is the perimeter, A is the projected load-bearing contact area, d is the penetration depth, and $R = 200\ \mu m$ is the scratch probe tip radius.

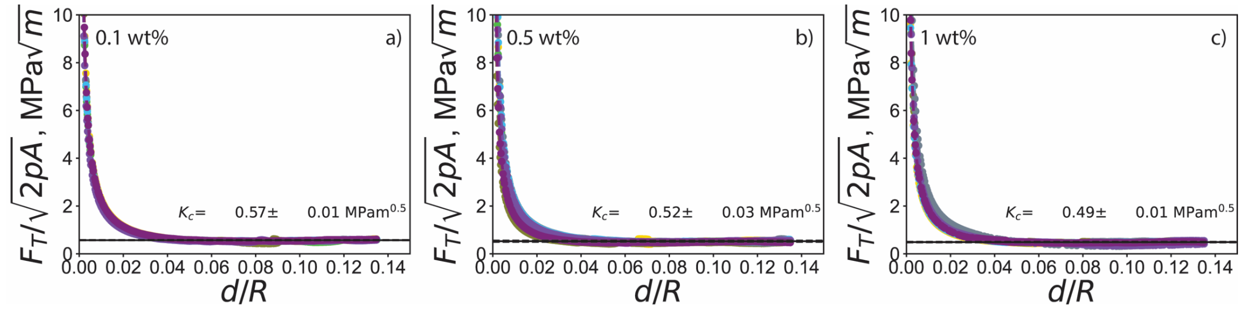

Figure S22: Scaling of scratch force for for KGP_PAN_WG. $F_T$is the horizontal force, p is the perimeter, A is the projected load-bearing contact area, d is the penetration depth, and $R = 200\ \mu m$ is the scratch probe tip radius.

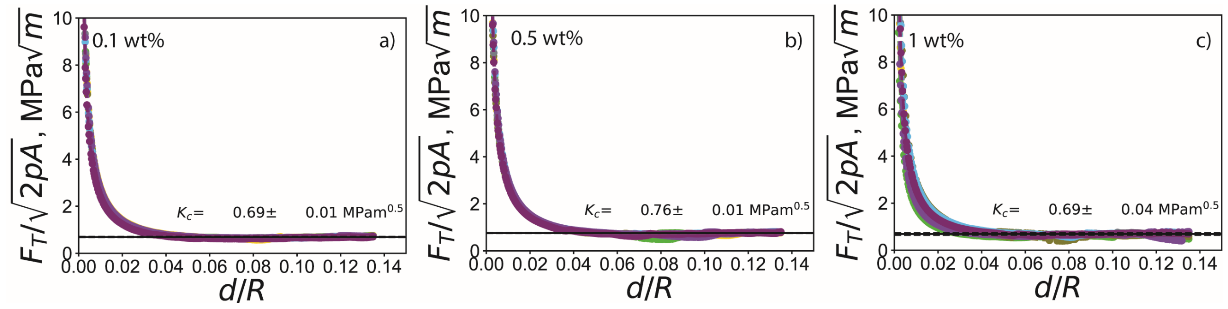

Figure S23: Scaling of scratch force for for KGP_PAN_WG. $F_T$is the horizontal force, p is the perimeter, A is the projected load-bearing contact area, d is the penetration depth, and $R = 200\ \mu m$ is the scratch probe tip radius.
